# Integrative Machine Learning and Immunoinformatics-Guided Design and In Silico Validation of a Multiepitope DNA Vaccine Against Avian Metapneumovirus

**DOI:** 10.3390/v18101084

**Published:** 2026-10-01

**Authors:** Fatma Nur Gazeyoglu, Abid Ullah Shah, Maged Gomaa Hemida

**Affiliations:** Department of Veterinary Biomedical Sciences, Lewyt College of Veterinary Medicine, Long Island University, Brookville, NY 11548, USA; fatma.gazeyoglu@my.liu.edu (F.N.G.); abidullah.shah@liu.edu (A.U.S.)

**Keywords:** avian metapneumovirus, aMPV, DNA vaccine, epitope mapping, machine learning, in silico prediction, molecular docking

## Abstract

Avian metapneumovirus (aMPV) is an emerging viral pathogen causing many outbreaks in chickens all over the world. There are several subtypes of the MPV circulating in chickens in the US. The current circulating subtypes of the virus in the US chicken are A, B, and C. Despite the availability of some aMPV vaccines in the US, most of these vaccines are based on foreign strains, particularly European aMPV-A/B strains. Their protective efficacy against contemporary U.S. aMPV isolates has not been fully established. The main goal of this study is to integrate the most recent aMPV genome sequencing data and the machine learning tools to design a novel aMPV. The machine learning tools such as epitope mapping, molecular docking, and immune simulation were used to design the multiepitope DNA vaccine based on the top-ranked epitopes of two major surface proteins of the virus (F and G). The top-ranked seventeen epitopes representing B cells, CD4 and CD8 epitopes were linked using linkers, with IL-18 added as an adjuvant. The selected epitopes showed high antigenicity, no toxicity and no allergenicity values among the screened epitopes. The molecular docking analysis of the designed vaccine construct showed a high binding affinity to the MHC class I and II epitopes to chicken alleles. The immune simulation analysis of this vaccine provides an in silico proof of concept assessment through modeling of the vaccine potential to elicit humoral and cell-mediated immunity. Further functional studies are required to test the immunogenicity and the efficacy of this novel vaccine before applications using chickens and turkey.

## 1. Introduction

Avian metapneumovirus (aMPV) is currently a neglected respiratory viral poultry pathogen, even in the US. The aMPV causes several clinical diseases in various species of birds, including swollen head syndrome in chickens and rhinotracheitis in turkeys [1]. Although the main tropism of the aMPV is a primary targeting of the respiratory system, the virus can do further damage to other tissues like the reproductive system. The aMPV infection may enhance the development of other infections in birds, such as colibacillosis, causing serious damage to infected chickens [2]. The aMPV is classified under the family *Pneumoviridae* and genus *Metapneumovirus*. The virus is subclassified into several subtypes [2]. Subtypes A and B are found mainly in Europe; subtype C in the United States, Canada, China, France, and South Korea; and subtype D is found only in France [1]. Subtype D has not been reported since its first discovery in 1985, while Australia remains the only continent considered aMPV-free. Subtype B remains generally the most prevalent subtype of the current groupings [3].

Due to a lack of monitoring of the virus at the genomic level, it has the ability to mutate and evolve, particularly within migratory birds. This hypothesis is supported by the most recent outbreak of aMPV subtypes A and B reported in the United States in 2024, where infection rates were extremely high and carried mortality rates of 30 to 50%. These rates were mostly attributed to the effects of secondary bacterial infections, with one exceptional case in North Carolina reporting mortality rates as high as 80% [4]. At the current time, there are no therapeutic interventions for aMPV and preventative control strategies rely heavily on diagnostic techniques such as RT-PCR and vaccination measures. This is because aMPV resembles other respiratory diseases such as Newcastle disease virus and avian influenza virus [5]. While vaccines for aMPV have been developed and used since the 1980s, continuous outbreaks point towards vaccine failures, with many vaccines coming in the form of live vaccines that research indicates can revert to a virulent form [1]. This points towards the urgency of the design and the development of a new generation of vaccines against aMPV in order to cope with the dynamic changes of the virus at the genomic level and to avoid vaccine escape and failure of the currently used vaccines. Ideally, the new generation of vaccines should be prepared against the currently circulating strains and subtypes of the virus in certain areas. Thus, the continuous monitoring of viruses, encompassing not only aMPV but also other human, bird and animal viral diseases, will provide a basic foundation for the design of any new generation of vaccines [6,7,8,9,10,11,12,13].

The genome of aMPV is a negative-sense RNA with eight genes encoding various proteins: nucleoprotein (N), phosphoprotein (P), matrix protein (M), fusion protein (F), second matrix protein (M2), small hydrophobic protein (SH), attachment protein (G), and large polymerase protein (L). Of the proteins, the F and G proteins are integral for virus attachment and entry, with the G protein playing a role in mediating the attachment to the host cell and the F protein promoting pH-independent fusion between the cell membrane and virus envelope [14].

The gene that encodes the G protein has an extensive history of genetic mutations—point mutations, insertions and deletions—leading to variations in size due to truncation of the G gene [15]. The protein stands out due to its unique sequence among other paramyxoviruses, where the hemagglutinin-neuraminidase (HN) attachment differs significantly [1]. The protein is heavily glycosylated with both N- and O-glycosylated sites, suggesting a reduced antigenicity through shielding of the virus protein with host-specified sugars [14].

On the other hand, the F protein is highly conserved and less variable than the G protein when comparing between the various subtypes of aMPV [16]. The native aMPV-F protein is present in an inactive form known as the F0 form, which is cleaved into two disulfide-linked subunits, F1 and F2, mediated by host cell proteases such as furin, which is critical for the viral fusion to the host cell membrane as well as for viral entry [1]. The F protein can bind to the cellular receptors and facilitate fusion with the virus and host cell membranes without the involvement of the G or SH proteins, while also having the capability to mediate receptor binding [5]. As a result of the need for a higher level of protection against aMPV, there have been several approaches to vaccine work, including live, inactivated, recombinant and DNA vaccines, with each variation having its advantages and drawbacks. The integration of the genome sequencing data of the currently circulating strains of the target viruses with the new development of machine learning, molecular docking and immune simulation has paved the way for the development of many vaccines against various viruses of humans/animals and birds [17,18,19,20,21]. In the current study we incorporated genome data and machine learning tools into the design and development of a vaccine for aMPV against this emerging viral pathogen [22,23,24,25].

The ability to rapidly screen datasets for the identification of epitopes, prediction of model structures, and visualization of ligand–receptor interactions using artificial intelligence shows great promise for vaccine development, changing the time and cost of development immensely [26,27,28,29]. The use of DNA vaccines is favorably looked upon, with the use of these types of vaccine constructs associated with low cost development, short synthesization time, and stability [30]. When combined, genome sequencing and machine learning tools provide strong tools for the development of next-generation vaccines with high levels of precision and accuracy [20,31,32]. The variety of vaccine designs of this nature pave the way for innovation and better vaccines for humans and animals.

The present study aimed to design a multiepitope DNA-based vaccine, using top-ranked B and T cell epitopes using two major proteins for aMPV (F and G). The designed DNA vaccine construct demonstrates a high antigenicity and strong binding simulation to chicken innate immune receptor (TLR7). In silico cloning as well as immune simulation were performed, showing great potential in stimulating an immune response through vaccination. The development of DNA vaccines has hampered the success of this type of vaccine for decades; however, the incorporation of DNA vaccines with nanoparticles has enhanced the delivery of the designed vaccines and been proven to produce robust immune response in the vaccinated hosts [33,34,35,36].

The current data provide an in silico blueprint in which, to validate the vaccine construct for application, further experimental studies will need to be performed. These experiments should be undertaken as part of in vivo studies and as part of studies on the vaccination of chickens and other birds, like turkeys and ducks, which are commonly susceptible to aMPV.

## 2. Materials and Methods

### 2.1. Multi-Epitope Vaccine Design Tools

A total of 25 computational platforms, software, and servers were used in the design, validation, and simulation of the proposed vaccine construct in silico. A comprehensive summary of the platforms used, including information on the training databases and host species origin, can be seen in Table 1.

### 2.2. Retrieval of Avian Metapneumovirus Protein Sequences and the Multiple Sequence Alignment

The amino acid sequences of the fusion (F) and attachment (G) proteins from various regions, such as the USA, South Korea, Brazil, China, and Netherlands, and obtained in the last 15 years were retrieved from the National Center for Biotechnology (NCBI) GenBank database (https://www.ncbi.nlm.nih.gov/protein/, accessed on 24 September 2026). Sequences from subtypes A, B, and C were included. Any sequences with ambiguous bases, partial sequences, and duplicate isolates were excluded. Isolates of the same sequence were reduced so as to not overpower the sequence alignment. In total, 53 sequences of the fusion protein and 68 sequences of the attachment protein were used to perform multiple sequence alignment (MSA) using Geneious v2026.1.2 (www.geneious.com, accessed on 24 September 2026) and Clustal Omega v1.2.2 (https://www.clustal.org/people-search/, accessed on 24 September 2026) with default parameters [37]. Supplementary File S1 provides all information about these sequences. The most conserved regions of the proteins were further considered for B cell and T cell (MHC class I and class II) epitope prediction. The Geneious software was then used to create a phylogenetic tree using PHYML, with 1000 bootstraps [38].

### 2.3. Mapping of B Cell Epitopes of the Fusion and Attachment Proteins in Avian Metapneumovirus

#### 2.3.1. Prediction of the Linear B Cell Epitopes

For the mapping of B cell epitopes using the generated consensus sequences for the two proteins (F and G), ABCpred was used (https://webs.iiitd.edu.in/raghava/abcpred/, accessed on 24 September 2026) [39]. The length of target epitopes was set to 16 and 18 mers with default parameters of 0.51 as the threshold. Epitopes were filtered by matching the predicted linear epitopes from the IEBD analysis resource server (https://tools.iedb.org/ellipro/, accessed on 24 September 2026) [40]. Further filtration of the epitopes was performed based on antigenicity, allergenicity, and toxicity. The antigenicity was assessed using the default threshold of 0.4 on the VaxiJen v2.0 server (http://www.ddg-pharmfac.net/vaxijen/VaxiJen/VaxiJen.html, accessed on 24 September 2026) [41]. Allergenicity was assessed using the AllerTOP v.2.1 server (http://ddg-pharmfac.net/AllerTOP/, accessed on 24 September 2026) [42]. The toxicity was assessed using the default settings of the ToxinPred server (https://webs.iiitd.edu.in/raghava/toxinpred/multi_submit.php, accessed on 24 September 2026) [43].

#### 2.3.2. Prediction of the Discontinuous/Conformational B Cell Epitopes

Discontinuous/conformational B cell epitopes were identified using IEDB’s prediction platform ElliPro (https://tools.iedb.org/ellipro/, accessed on 24 September 2026) [40]. The PDB files for the proteins were created using trRosetta (https://yanglab.qd.sdu.edu.cn/trRosetta/, accessed on 24 September 2026) [44]. The default settings were used with 0.5 as the minimum score and 6 angstrom for the maximum distance. Visualization of the epitopes was completed using Pymol version 2.5.4.

### 2.4. Mapping of the T Cell Epitopes of the Fusion and the Attachment Proteins of Avian Metapneumovirus

Prediction of Cytotoxic T Cell Epitopes (MHC Class I)

The cytotoxic T cell epitopes were selected based on the chicken MHC class I peptide-binding motifs in accordance with the BF2*4, BF2*12, BF215, and BF2*19 haplotypes or BF2∗02:01/BF2∗04:01/BF2∗21:01 as previously described [45,46]. Protein sequences were screened using the following patterns: x-(D or E)-x-x-(D or E)-x-x-E, x-x-x-x-(V or I)-x-x-V, x-x-x-x-(V or I)-x-x-x-V, (K or R)-R-x-x-x-x-x-Y, (K or R)-R-x-x-x-x-x-x-Y, x-R-x-x-x-x-x-(Y,P,L,F), x-R-x-x-x-x-x-x-(Y,P,L,F), and x-A/V/I/L/P/S/G-x-x-x-x-x-x-V/I/L. The epitopes matching the patterns were screened for antigenicity, allergenicity, and toxicity as previously described [41,42,43].

Prediction of the Helper T Cell Epitopes (MHC Class II)

Due to the lack of information about the chicken alleles in the IEDB database, the best human allele surrogates were used for MHC class II epitope prediction. Through the analysis of heatmap data in the MHCcluster—as done in previous work indicating a high degree of similarity between the used human/chicken alleles, particularly in the peptide binding grooves—the best substitute alleles for MHC class II were found to be DRB1:1310, DRB1:1366, DRB1:1445, and DRB1:1482 [47]. The helper T cell epitopes were identified using the IEDB server (https://tools.iedb.org/mhcii/, accessed on 24 September 2026) [48]. The binding predictor NetMHCII pan 4.1 BA (recommended binding predictor: 2023.09) was used, sorting the peptides in accordance with the inhibitory concentration (IC50) and percentile rank [49]. Peptides with an IC50 < 50 nM and percentile rank < 4 were chosen and further filtered via antigenicity, allergenicity, and toxicity as previously described [41,42,43]. MixMHC2pred (http://mixmhc2pred.gfellerlab.org/, accessed on 24 September 2026), using the MHC class II alleles Gaga_BLB1_002_01, Gaga_BLB1_012_01, Gaga_BLB2_002_01, Gaga_BLB2_012_01, Gaga_BLB2_012_02, was then used to predict the chicken alleles associated with the peptides [50]. The use of the predictor is solely to evaluate the compatibility of the identified chicken alleles with the predicted epitopes.

### 2.5. Molecular Docking of Predicted T Cell Epitopes to Chicken MHC-I and MHC-II Alleles

The chicken MHC allele sequence files (BF and BL) were retrieved from the Protein Data Bank (PDB). Molecular docking of the predicted MHC-I and MHC-II to the respective alleles was conducted using HDOCK (http://hdock.phys.hust.edu.cn/, accessed on 24 September 2026) [51]. Epitopes were filtered based upon their docking ability. The Pymol software was used to visualize the key residues, as well as information about the hydrogen bond donors and acceptors.

### 2.6. Assessment of the Top-Ranked Epitopes Affinity to the Chicken Genome and Membrane Topology Prediction

The full amino acid sequence of the chicken (*Gallus gallus*) genome was retrieved from the National Center for Biotechnology (NCBI) GenBank database (https://www.ncbi.nlm.nih.gov/protein/, accessed on 24 September 2026) [52]. The sequence was then uploaded to Geneious v2026.1.2 (www.geneious.com, accessed on 24 September 2026). The top-ranked epitopes for B and T cells were assessed for similarity to the chicken genome to prevent any unwanted autoimmune reaction to the designed vaccine. The predicted epitopes were mapped onto their respective protein structures using Pymol to assess surface exposure and estimation of the accessibility of the epitopes to antibody recognition was done using solvent-accessible surface area (SASA) analysis. Top-ranked epitopes were also assessed for conservation against the selected isolates for the project. The sequences of the F and the G proteins were then added through the membrane-topology prediction server DeepTMHMM (https://dtu.biolib.com/DeepTMHMM, accessed on 24 September 2026) as well as the Expasy ProtScale Tool (https://web.expasy.org/protscale/, accessed on 24 September 2026) using the Kyte–Doolittle algorithm. Through this analysis, top-ranked B cell epitopes were screened to make sure no epitopes were selected that are hydrophobic or located within the transmembrane domains or cytoplasmic tail regions.

### 2.7. Assembly of Multi-Epitope Vaccine Using Top-Ranked Epitopes

The vaccine sequence was arranged starting with the Kozak sequence followed by a start codon. The primary area of the vaccine comprises the top-ranked B and T cell epitopes based on the filtering values such as antigenicity, allergenicity, toxicity, molecular docking, and alignment to host genome. Linkers such as KK, GPGPG, and AAY were used to link the epitopes. Towards the end of the C-terminal end of the vaccine, chicken IL-18 is linked (Accession No. CAB96214) as an adjuvant, as well as EAAK linker [53,54]. Prior to the stop codon, a 6 x His-tag (H) tag is added to the construct for the expressions identification of the vaccine.

### 2.8. Analysis of Mulit-Epitope Vaccine Disulfide Bond Engineering

For the stability of the protein, it is essential to create disulfide bonds between the cysteine residues. The vaccine protein was submitted to Disulfide by Design v2.12 web server (https://cptweb.cpt.wayne.edu/DbD2/, accessed on 24 September 2026) for disulfide bridging [55]. The X^3^ value and the Cα-Cβ-Sγ angle were kept as the default parameters. Based upon previous studies, the X^3^ value must range between −87 and +97◦, while the energy score must not exceed 2.2 kcal/mol for the disulfide bridging. For bridging between possible residue pairs, mutations to cysteine residues occurred using the server’s “Create/View Mutant” option.

### 2.9. Assessment of the Physiochemical Properties and Stability of the Designed Multi-Epitope Pan-Avian Metapneumovirus Vaccine

The designed vaccine was assessed for its physiochemical properties using Protparam server (https://web.expasy.org/protparam/, accessed on 24 September 2026) [56]. The antigenicity was assessed using the default threshold of 0.4 on the VaxiJen v2.0 server (http://www.ddg-pharmfac.net/vaxijen/VaxiJen/VaxiJen.html, accessed on 24 September 2026) [41]. Allergenicity of the vaccine was assessed using the AllerTOP v.2.1 server (http://ddg-pharmfac.net/AllerTOP/, accessed on 24 September 2026) [42]. The solubility of the vaccine construct was determined using the Innovagen peptide solubility calculator (https://pepcalc.com/peptide-solubility-calculator.php, accessed on 24 September 2026). In terms of the stability and physical mobility of the atoms, the vaccine construct was analyzed using normal mode analysis (NMA) using the iMODS server tool (https://imods.iqf.csic.es/, accessed on 24 September 2026) [57]. Multiple descriptors such as eigenvalues, covariance, B-factors, and deformability and intrinsic motions of multiplex proteins were analyzed. Eigenvalues were used to assess motif stiffness and the biological targets’ efficacy measurement was used to predict deformity of the main chain.

### 2.10. Predictions of the Secondary and Tertiary Structures of the Multi-Epitope DNA Vaccine

The PDB file for the vaccine was created using trRosetta (https://yanglab.qd.sdu.edu.cn/trRosetta/, accessed on 24 September 2026) [44]. The secondary structure of the vaccine was predicted using PSIPRED (https://bioinf.cs.ucl.ac.uk/psipred/, accessed on 24 September 2026) [58]. The tertiary structure of the vaccine was predicted using AlphaFold Server (https://alphafoldserver.com/, accessed on 24 September 2026) [59]. The ProSA-web server (https://prosa.services.came.sbg.ac.at/prosa.php, accessed on 24 September 2026) was used to assess the quality of the model [60]. The stability of the construct was analyzed using Ramplot (ramplot.in) [61].

### 2.11. Molecular Docking of Designed Multi-Epitope Vaccine to Chicken Toll-like Receptor (TLR)

Chicken Toll-like receptor (TLR) was selected to be used for molecular docking with the vaccine construct. The chicken TLR7 (UniProt ID: A0A1L4FML6) was retrieved from Uniprot and the molecular docking was conducted using HDOCK (http://hdock.phys.hust.edu.cn/, accessed on 24 September 2026), showing the structures of the two molecules as well as the docking interactions [51]. Visualization of the docking was done by the Pymol server. A detailed analysis of the binding interfaces was characterized by the Tamarind Bio PDBsum tool (https://app.tamarind.bio/app, accessed on 24 September 2026) [62].

### 2.12. In Silico Immune Simulation of Designed Multi-Epitope Vaccine

To model the immune response to vaccination, the C-ImmSim server (https://kraken.iac.rm.cnr.it/C-IMMSIM/index.php, accessed on 24 September 2026) was used [63]. The use of the server is to simulate the injection of the vaccine construct, modeling the three major immune organs (bone marrow, thymus, and spleen) and the influence of the construct on the B and T lymphocytes. The default parameters were used with 1 injection of 20 mL volume and a duration of 200 simulation steps using a random seed. Every step of the simulations is indicative of eight hours of real time. An additional simulation of the vaccine without the IL-18 adjuvant was also done to see the difference between adding or not adding the adjuvant. The amino acid sequence of the vaccine was converted into a nucleotide sequence using VectorBuilder’s codon optimization tool (https://en.vectorbuilder.com/tool/codon-optimization.html, accessed on 24 September 2026). The sequence was inserted into the vector pcDNA3.1(+) with no overhangs accessed from the SnapGene website (https://www.snapgene.com/plasmids/image_consortium_plasmids/pcDNA3.1(%2B), accessed on 24 September 2026) using the insertion cloning action.

## 3. Results

### 3.1. Resuts of the Analysis of the Currently Circualting aMPV-F and G Protein Strains

**Figure 1:**
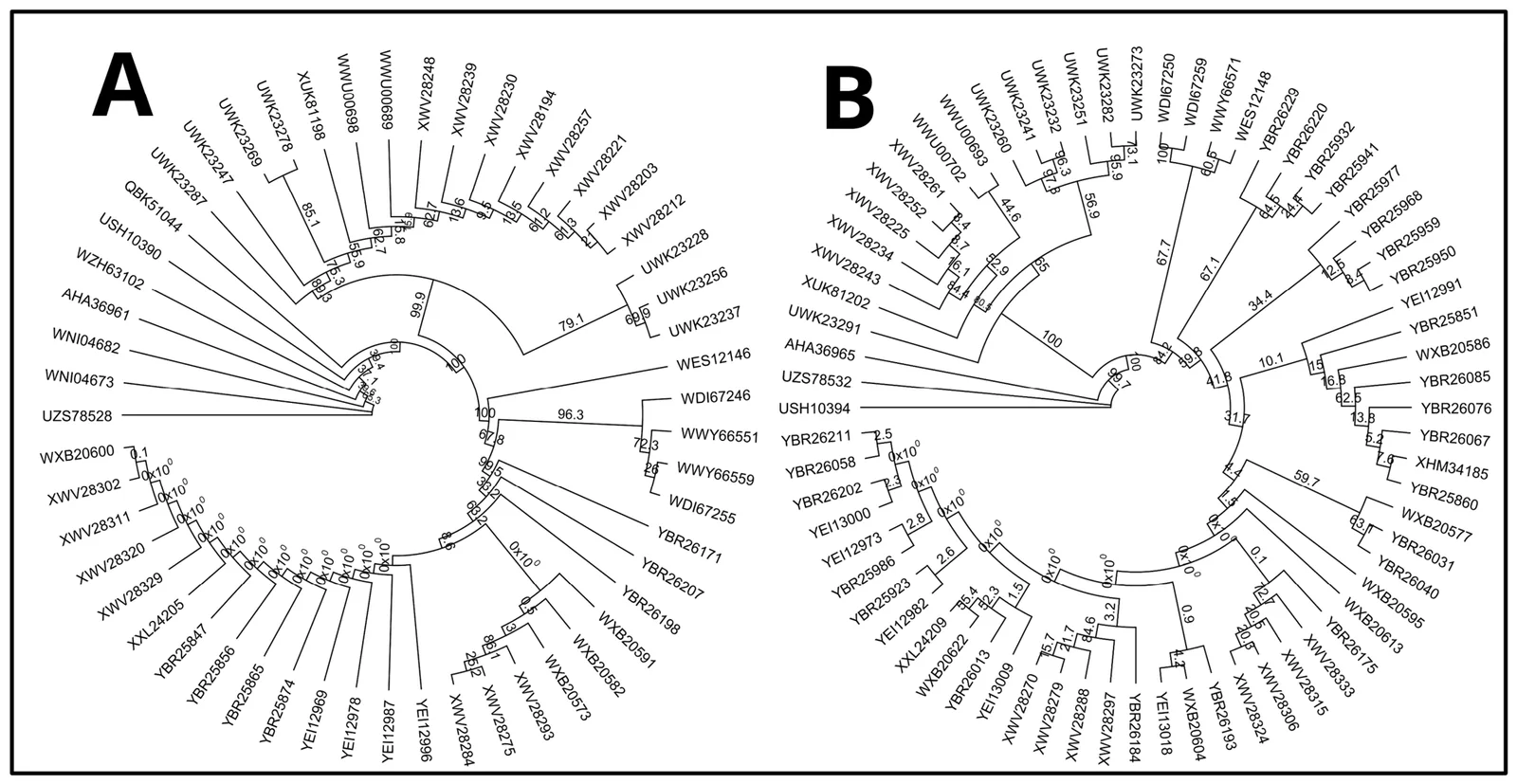
Phylogenetic analysis of the currently circulating strains of the aMPV/F/G-based proteins in chickens in the USA. (**A**) Fifty-three recent aMPV-F isolates; (**B**) phylogenetic tree analysis of selected 68 aMPV-G protein isolates.

Protein sequences of the F and G proteins in aMPV were extracted to perform multiple sequence alignment in Geneious. Sequences feature various avian species such as chickens, turkeys, and ducks. The list of the sequences used, and sample information can be found in Figure 1. In the analysis of the multiple sequence alignment, similarity levels can be seen as 87.2% for F protein and 66.1% for G protein. This indicates a higher level of genetic similarity in the F protein sequence among avian hosts than the G protein sequence.

### 3.2. B Cell Epitope Prediction and Evaluation Among the F and G Proteins of the Currently Circaulting aMPV Subtypes

The humoral immune response elicited by B cell epitopes has become a major factor in vaccine development. Linear/continuous B cell epitopes were predicted for aMPV proteins using the ABCpred computational tool. For the F protein, 54 epitopes were predicted and 46 epitopes were predicted for the G protein. All of the predicted epitopes were filtered for antigenicity, allergenicity, and toxicity and were matched to the predicted linear epitopes from the IEDB Ellipro tool. The remaining epitopes are shown in Table 2.

**Figure 2:**
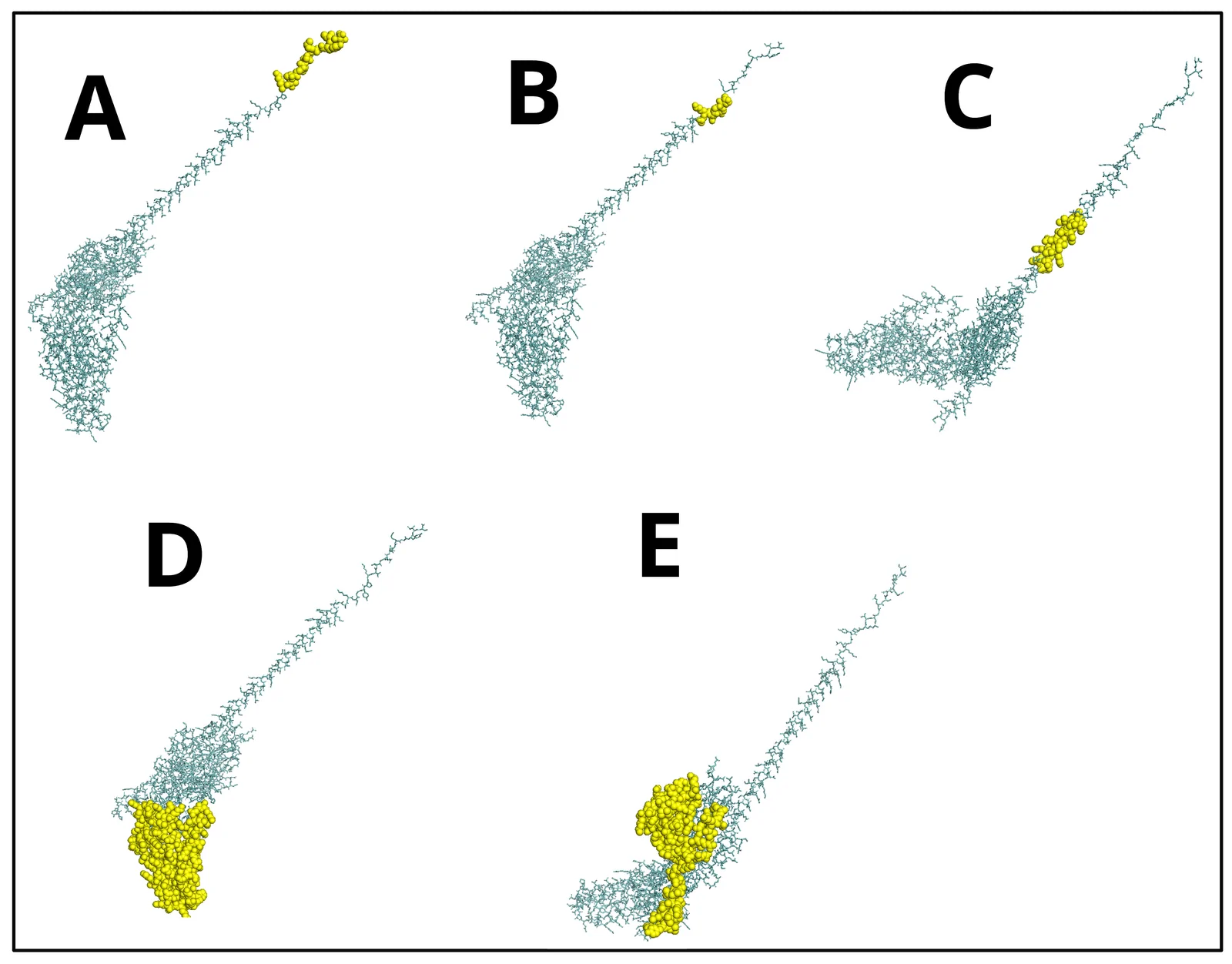
(**A**–**E**) The predicted conformational/discontinuous structures of the selected epitopes for the aMPV-F protein, visualized by the Pymol.

**Figure 3:**
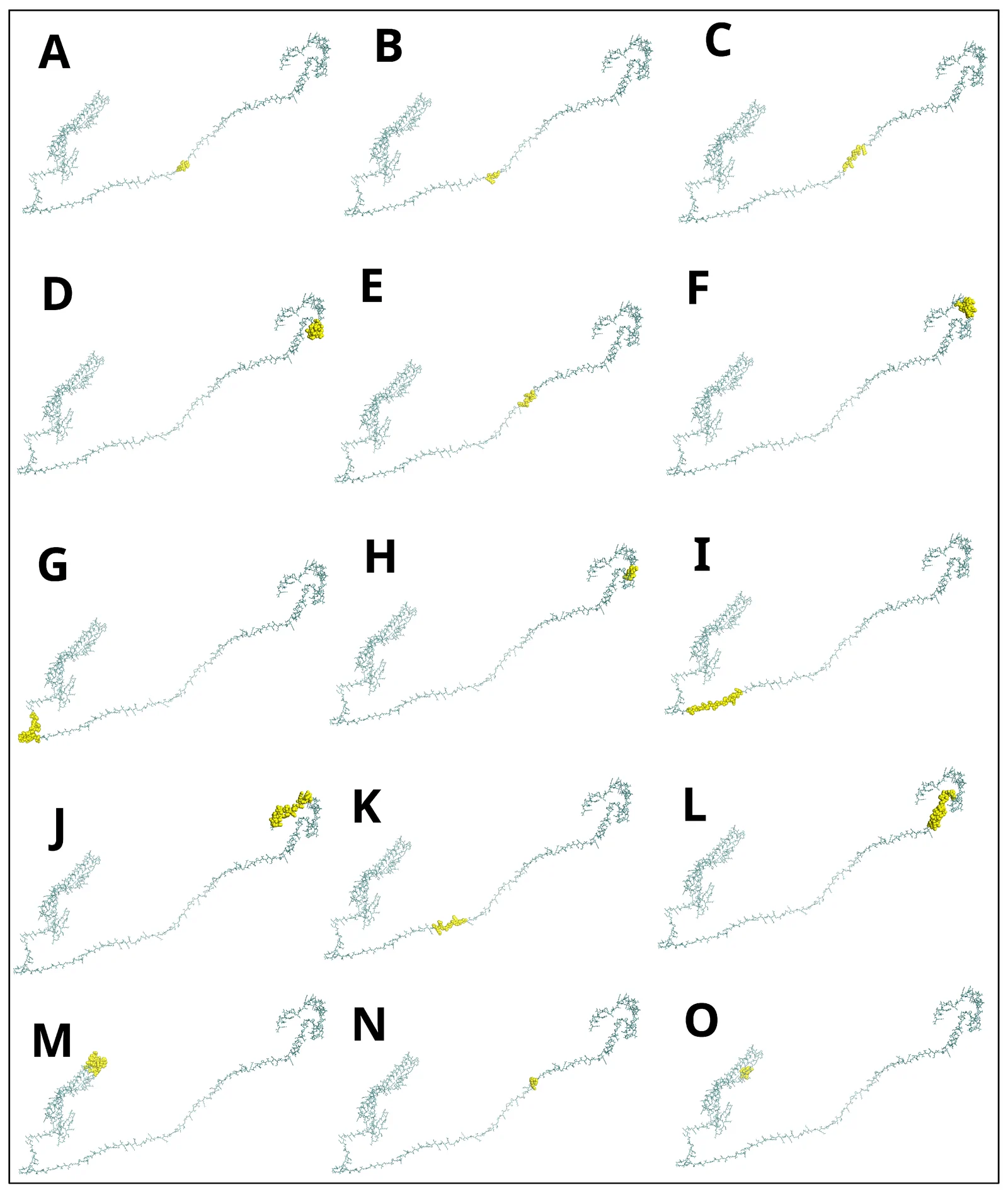
(**A**–**O**) The list and structure of the predicted conformational/discontinuous structures of the epitopes of the aMPV-circulating strains based on the G protein, visualized by the Pymol.

From the Ellipro server, the predicted conformational/discontinuous epitopes were also predicted using the 3D structure of the proteins, using the default settings of a minimum score of 0.5 and a minimum distance of 6Å. The respective epitope predictions for each protein can be found in Table 3. Visualization of the epitopes can be found in Figure 2 and Figure 3.

### 3.3. Cytotoxic T Lymphocyte (MHC Class I) and Helper T Lymphocyte (MHC Class II) Epitope Predictions and Assessments

Cytotoxic and helper T cells are important when signaling and fighting infection at the cellular level. In Table 4, the predicted MHC class I epitopes based upon the published patterns are listed. All epitopes were screened for antigenicity, allergenicity and toxicity. For cytotoxic T lymphocytes, 27 epitopes were found for F protein, and 21 epitopes were found for G protein.

In Table 5, the predicted MHC class II epitopes are listed with the corresponding binding affinity (IC50 < 50 nM) and per rank % from the IEDB NetMHCII pan 4.1 BA server for the aMPV proteins. Each of the epitopes were screened for antigenicity, allergenicity and toxicity. For the F protein, three helper T lymphocytes were found and, for the G protein, one epitope was found. The epitopes were then used in the MixMHC2pred to predict the associated chicken allele.

### 3.4. Assessment of the Molecular Docking of MHC Class I and II Epitopesof the Currently Circualting aMPV Strains with Chicken Alleles

**Figure 4:**
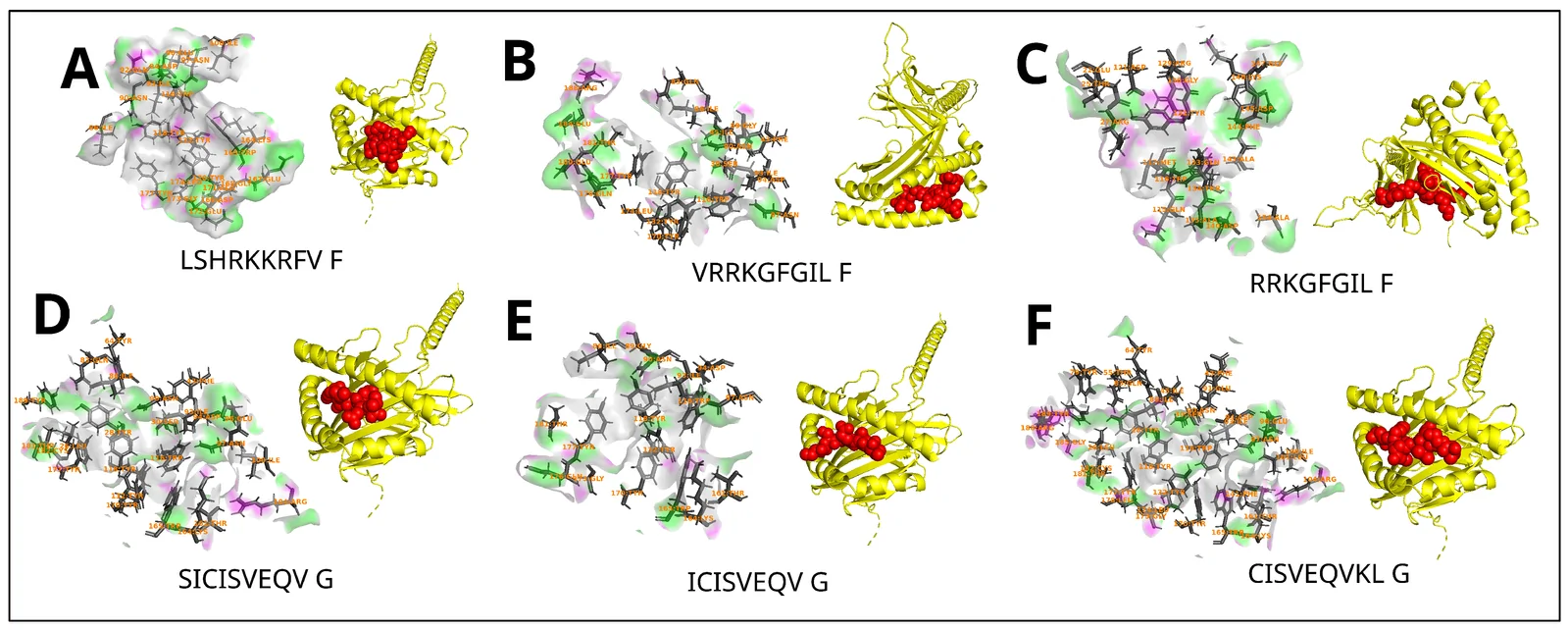
Results of the docking analysis between the predicted MHC class I chicken allele BF2 and the selected aMPV epitopes: (**A**) LSHRKKRFV F, (**B**) VRRKGFGIL F, (**C**) RRKGFGIL F, (**D**) SICISVEQV G, (**E**) ICISVEQV G, and (**F**) CISVEQVKL G.

**Figure 5:**
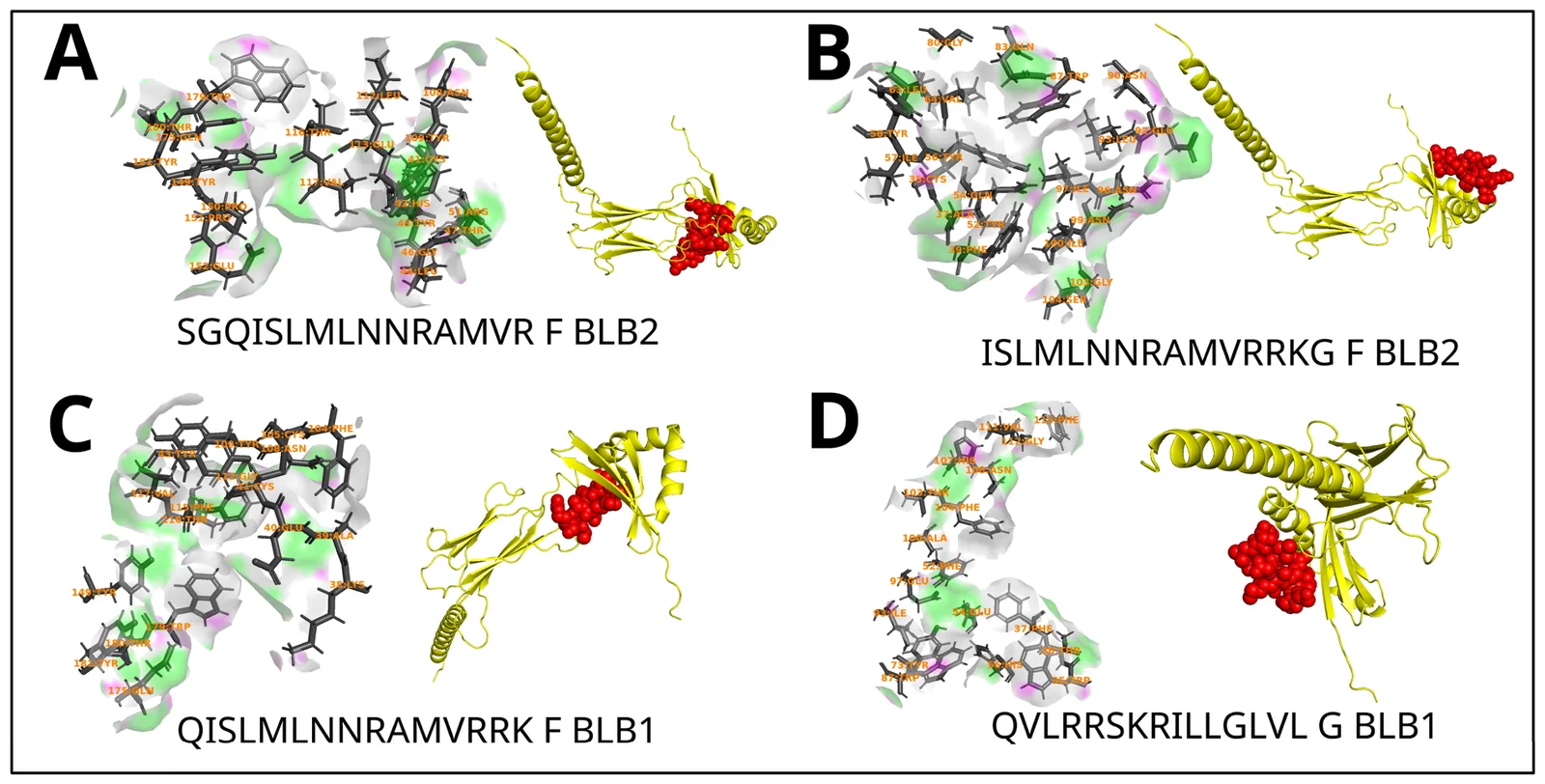
Results of the docking analysis between MHC class II chicken alleles (BLB1/BLB2) and selected epitopes: (**A**) SGQISLMLNNRAMVR F BLB2, (**B**) ISLMLNNRAMVRRKG F BLB2, (**C**) QISLMLNNRAMVRRK F BLB1, and (**D**) QVLRRSKRILLGLVL G BLB1.

Using the Hdock server, molecular docking analysis was performed between MHC class I and II epitopes and chicken alleles (BF2 for MHC class I and Gaga_BLB1 and Gaga_BLB2 for MHC class II). On the server the chicken alleles were set as the receptors and the predicted epitopes from Table 3 and Table 4 were set as the ligands. In Supplementary Excel Files S3 and S4, the binding affinity and confidence scores of the epitopes were listed. Visualization of the docking can be seen in Figure 4 and Figure 5, where H-bond donors can be seen highlighted in magenta, and H-bond acceptors can be seen highlighted in green.

### 3.5. Vaccine Construct Design

**Figure 6:**
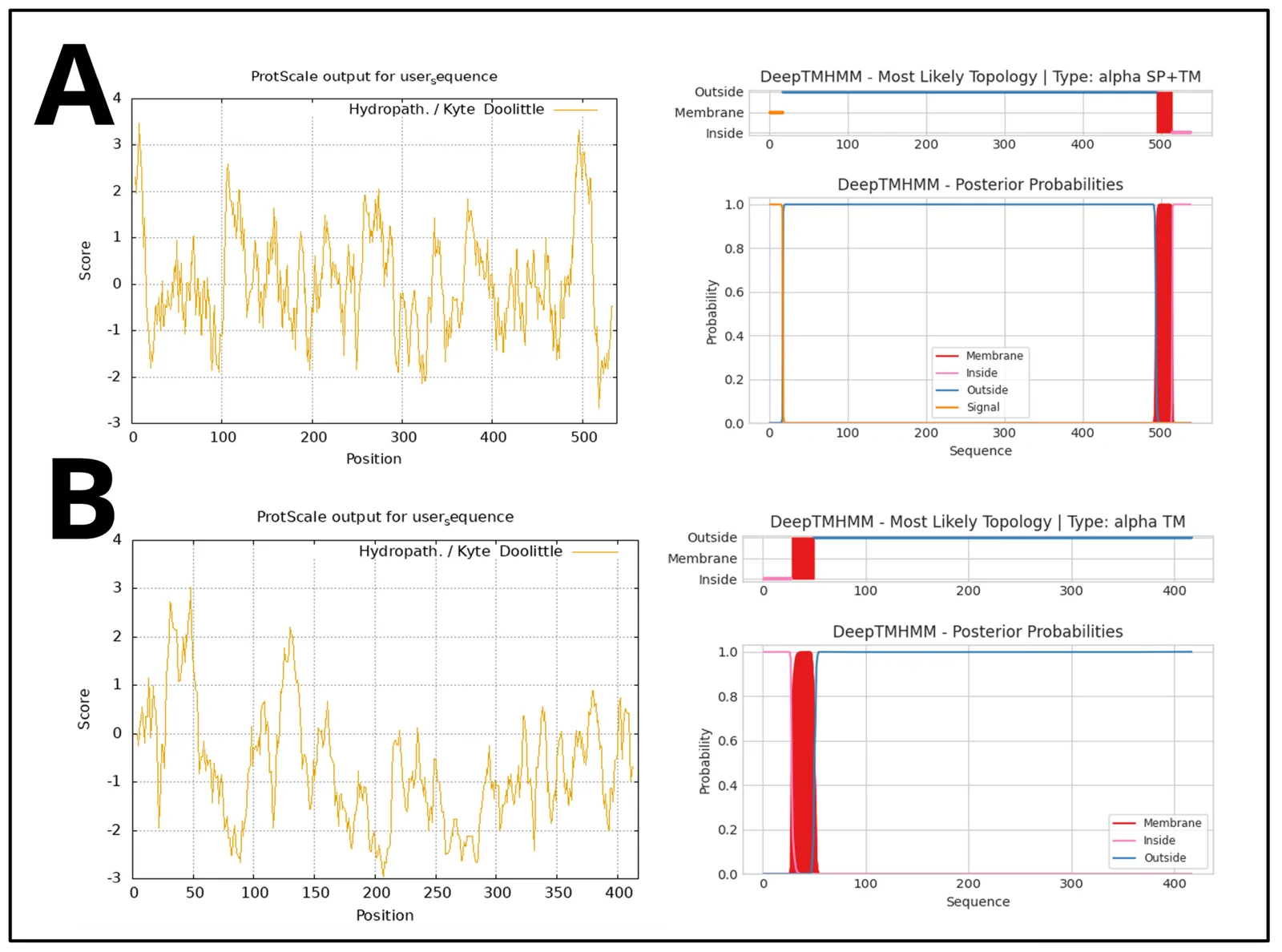
Results of the hydropathy and DeepTMHMM topology predictions for (**A**) F protein and (**B**) G protein.

**Figure 7:**
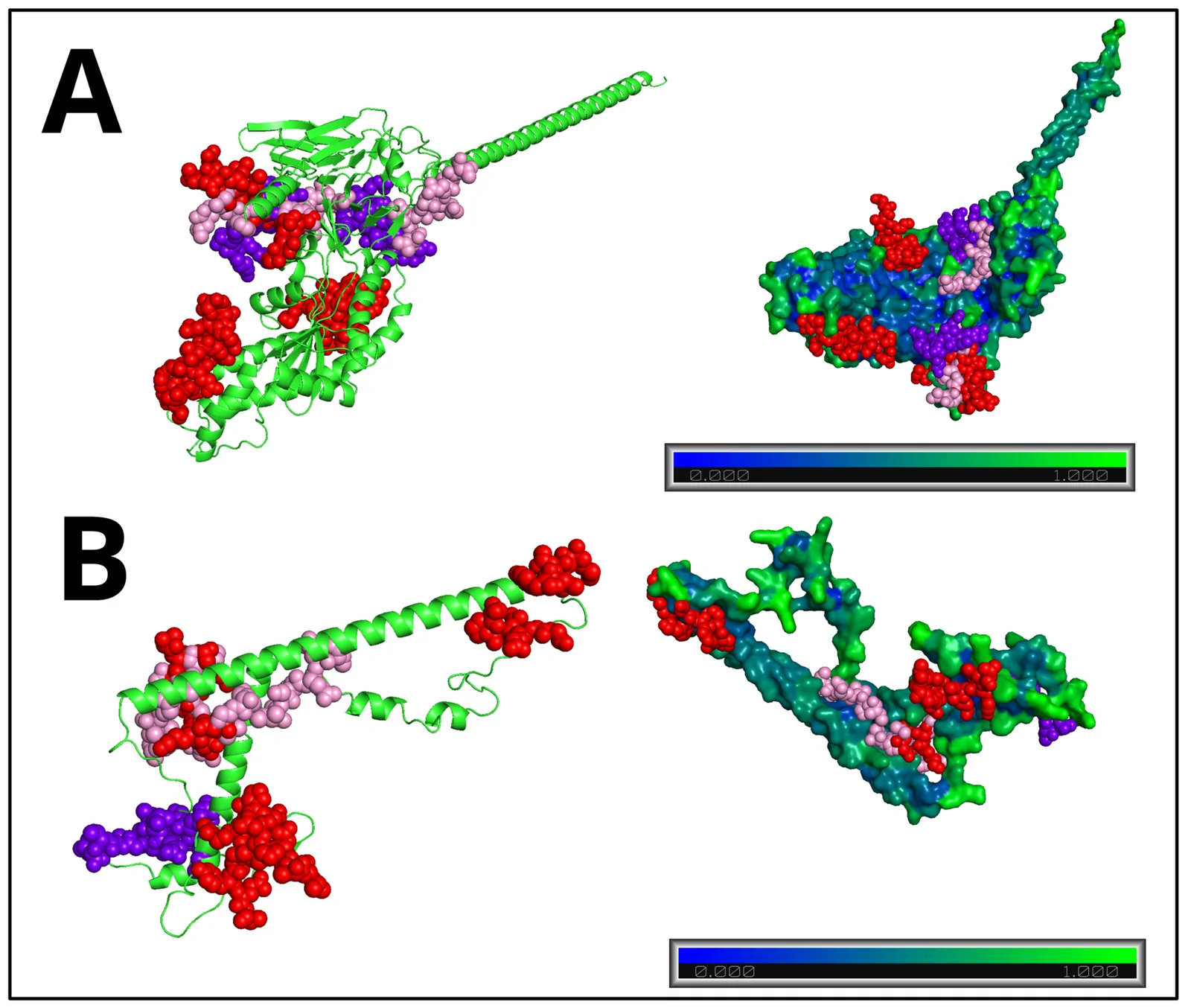
Epitope mapping and solvent accessibility analysis of the viral protein in aMPV: (**A**) F protein with the predicted B cell, MHC class I, and MHC class II epitopes mapped to the protein surface and the corresponding solvent accessible surface; (**B**) G protein with the predicted B cell, MHC class I, and MHC class II epitopes mapped to the protein surface and the corresponding solvent accessible surface.

To design the final vaccine construct, the top-ranked B cell epitopes and MHC class I and II T cell epitopes were chosen. The epitopes were selected based upon the antigenicity scores, non-allergenicity, non-toxicity, and binding affinity scores when applicable. In Figure 6 the hydropathy predictions and DeepTMHMM topology predictions for the F and G proteins were completed to also narrow the selection of the epitopes. All epitopes were also screened against the chicken amino acid genome for any matches. Of the B cell epitopes, the highest percentage of matching was 43.75%, 75.00% for CTL, and 40% for HTL. This indicated an unlikely chance for the vaccine construct to cause autoimmune interactions. The epitopes were then also matched against the circulating strains of aMPV used originally to see the individual sequences, subtypes, hosts, and geographical regions, seen in Supplemental File S5. In Figure 7, the mapping of the selected B cell, MHC class I, and MHC class II are shown in their respective docking locations as well as the solvent accessibility surface (SAS) regions (Table 6).

**Figure 8:**
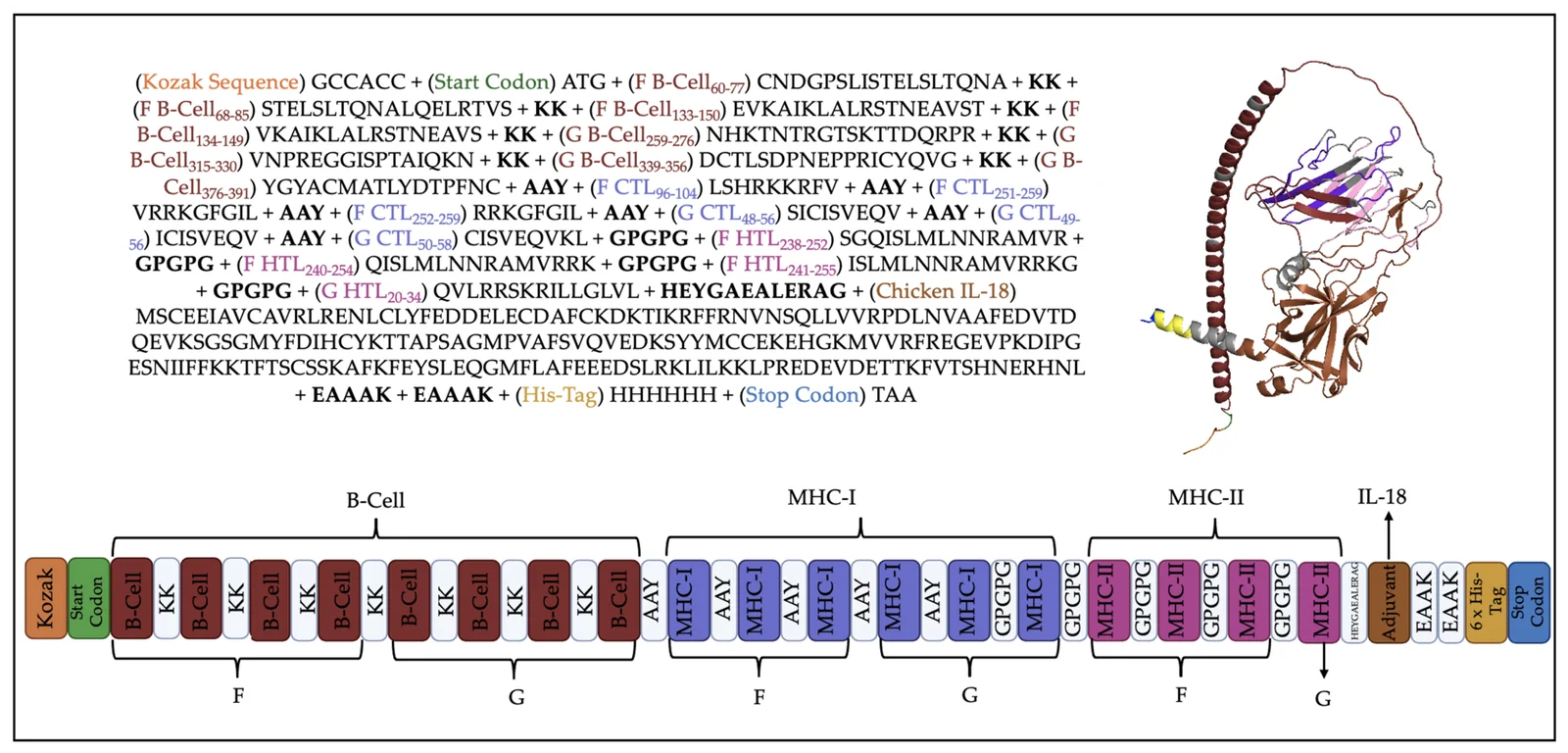
The schematic and the nucleotide sequences of the designed multiepitope aMPV-DNA vaccine construct using top-ranked epitopes.

The epitopes were linked using linkers such as KK, AAY, and GPGPC as well as the addition of the full-length chicken IL-18 gene (Accession No. CAB96214) as an adjuvant with a PEAK linker at the C terminal end of the construct. At the C-terminus for purification and identification of the vaccine expression, a 6 X His-tag (HHHHHH) is attached. For the construct as seen in Figure 8 the Kozak sequence in orange starts, followed by the start codon in green, while B cell epitopes are shown in red with a KK linker, MHC class I T cell epitopes are shown in purple with a AAY linker, and MHC class II T cell epitopes are shown in pink with a GPGPC linker. The MHC class II is linked to the IL-18 adjuvant in brown using HEYGAEALERAG while IL-18 is linked to the 6 x His-Tag in yellow using EAAAK followed by the stop codon in blue. In Table 6 all the selected epitopes and their information are listed.

### 3.6. The Disulphide Bond Engineering Results

**Figure 9:**
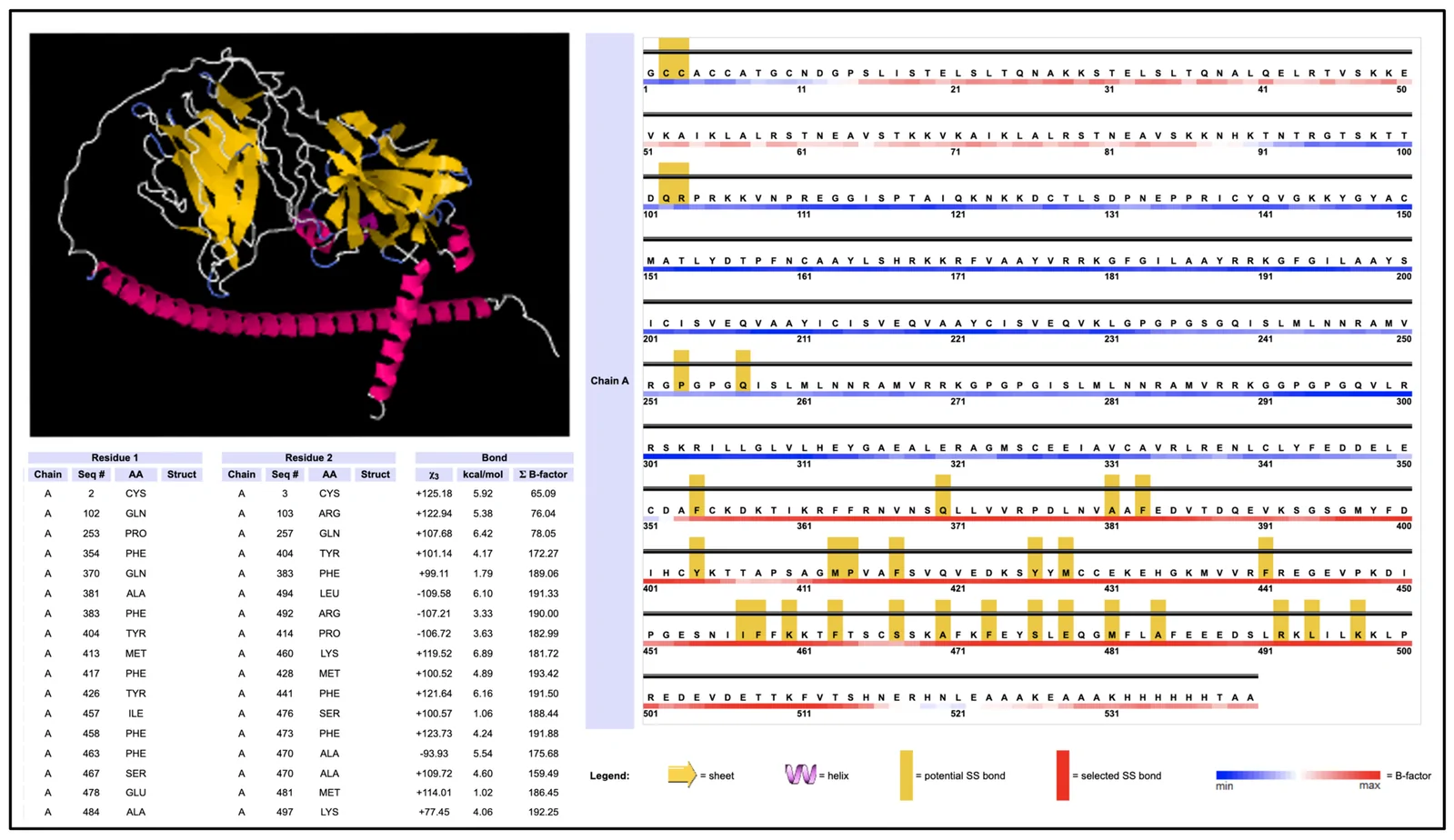
Analysis of the stability of the designed vaccine construct following disulfide bond engineering, highlighted regions in yellow represent prospective bonding sites.

The analysis from the Disulfide by Design v2.12 webserver showed one residue pair of GLY-ASP observed based upon the chi3 and B-factor energy parameters. The formation of the disulfide bonds was initiated with the introduction of cysteine residues, evaluating within the range of −87 to +97 chi3 values as well as an energy value < 2.2. Figure 9 shows the steps by which to identify the residues for the formation of disulfide bonds to enhance the stability of the vaccine construct. In the figure, none of the prospective bonds fall within the range.

### 3.7. Results of the Physiochemical Properties of the aMPV-DNA Vaccine Construct

The weight of the vaccine construct is predicted to be 59,937.17 Dalton with a theoretical isoelectric point of 9.34, indicative of an alkaline state of the vaccine. In total there are 57 negatively charged residues and 84 positively charged residues. The extinction coefficient measured at 280 nm in water was seen to be 26,580 with the assumption that all pairs of Cys residues form cystines. The aliphatic index was predicted to be 76.80, with a hydrophilic value of −0.384.

**Figure 10:**
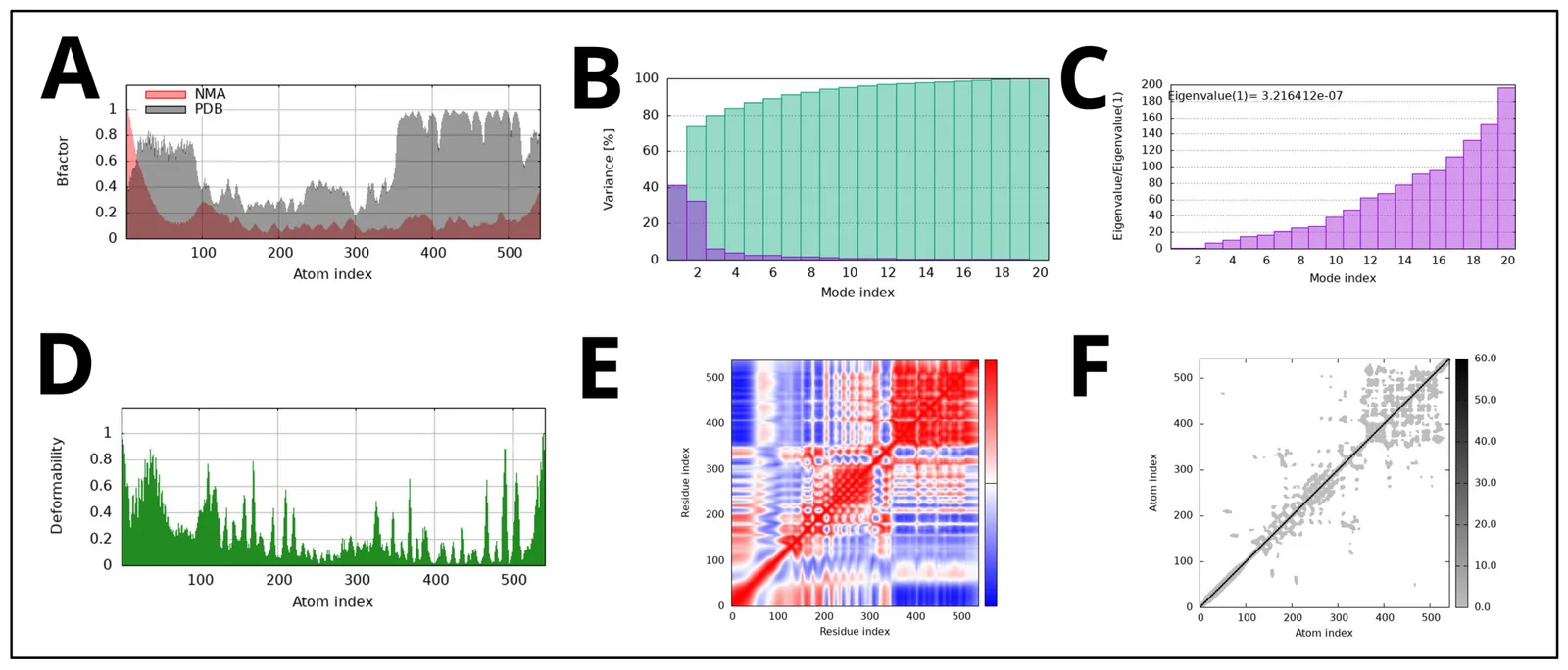
The vaccine protein normal mode analysis: (**A**) B-factor-NMA mobility, (**B**) variance (purple indicating individual variance, and green indicating cumulative variation), (**C**) eigenvalues, (**D**) deformability, (**E**) covariance map (correlated motions in red, uncorrelated motions in white, and anti-correlated motions in blue), and (**F**) elastic network.

Using the iMODS server tool, the stability and physical movement of the vaccine construct was analyzed with normal mode analysis (NMA). In the figure, the B factor in 10a is representative of the atomic fluctuations within the normal mode analysis when compared with that of the experimental mode of vibration, with values <50 indicating stable proteins and values >50 indicating flexible proteins. In 10b, the variance plot shows the restricted motion through green and purple colors reflective of the cumulative variance. In Figure 10C the eigenvalue of the complex was seen to be 3.216412 × 10^−7^. Figure 10D is the deformability plot, showing the capability of bending during motion while 10e is the covariance map showing the motion correlations, with red indicating correlated movements, blue indicating anti-correlated movements, and white indicating no movement. Lastly, in Figure 10F, the elastic network shows the stable regions with dense springs and flexible regions with sparse ones.

### 3.8. Results of the Secondary and Tertiary Structures of the aMPV Vaccine Construct

**Figure 11:**
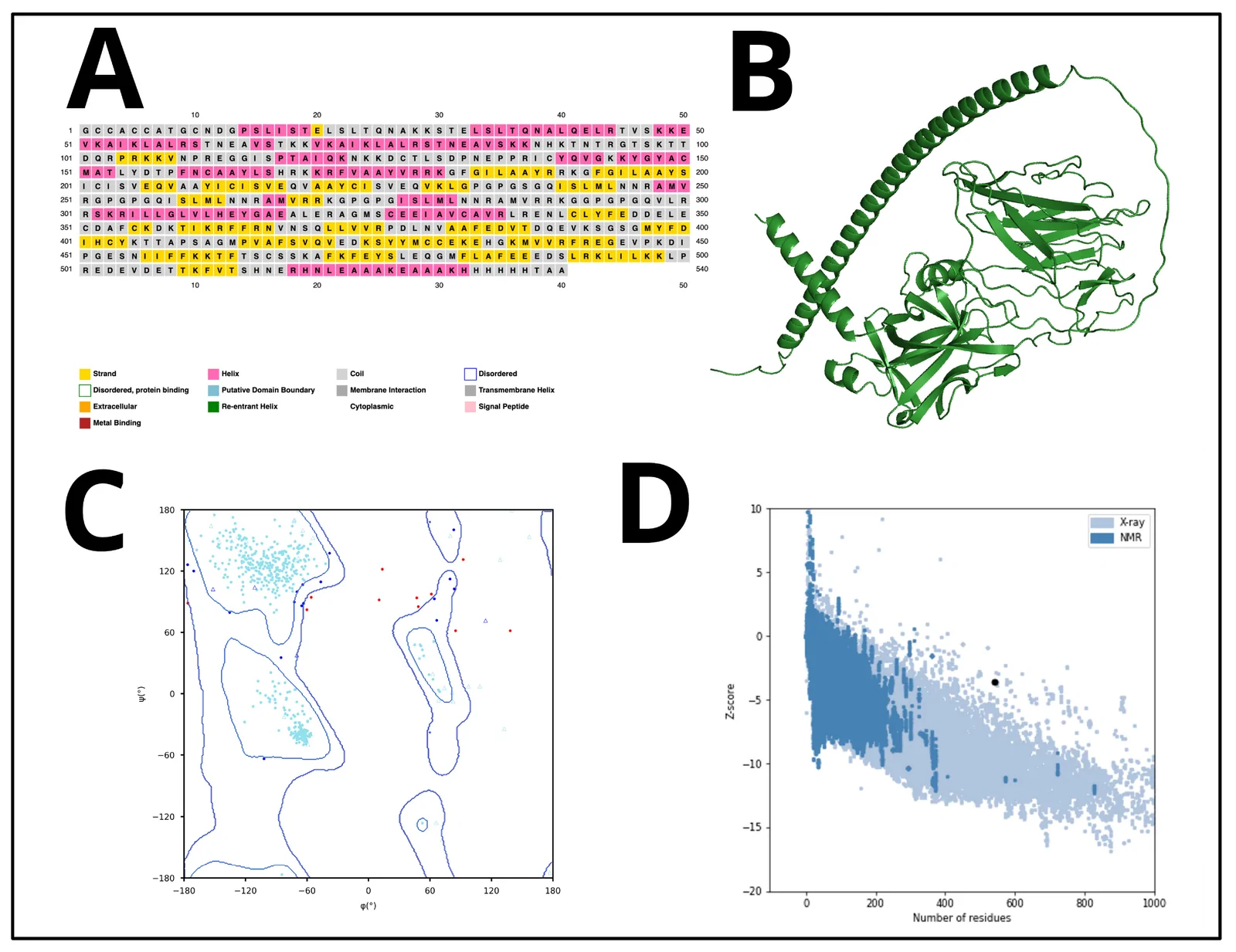
Structural analysis of the multiepitope vaccine construct for aMPV: (**A**) secondary structure prediction of the vaccine construct; (**B**) the 3D structure predicted using AlphaFold; (**C**) Ramachandran plot analysis conforming stability with a high number of blue dots in the respective regions; (**D**) overall model quality of vaccine construct as analyzed by the ProSA server with an overall Z-Score of −2.56.

The secondary and tertiary structures of the multi-epitope vaccine were analyzed and modeled through PSIPRED and AlphaFold. Evaluation of the quality of the structures were done using ProSA and Ramplot. All of these can be seen in Figure 11.

### 3.9. Molecular Docking of the Designed aMPV Vaccine Construct with the Chicken Toll-like Receptor (TLR7) Analysis

**Figure 12:**
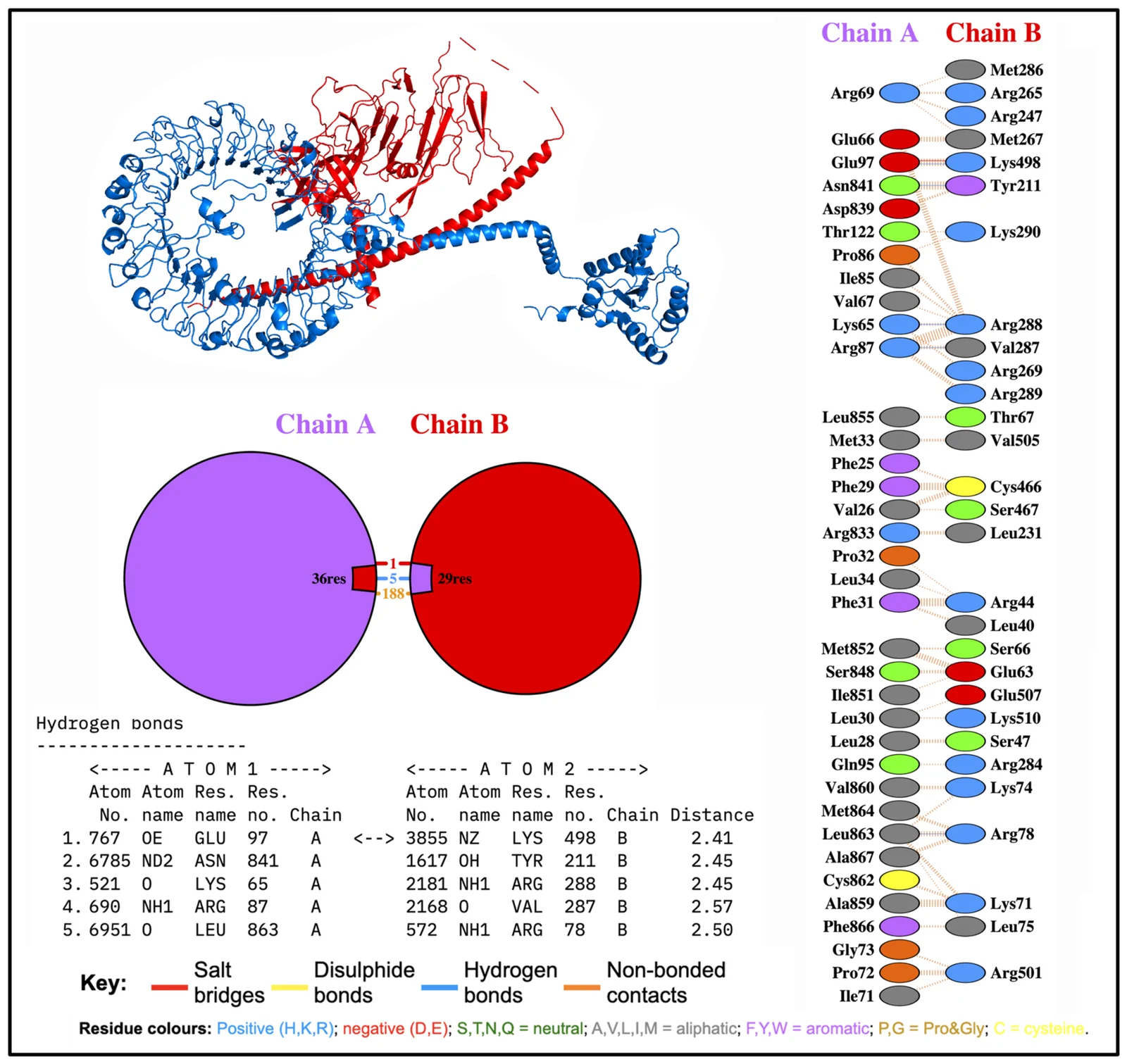
Results of the molecular docking analysis between chicken TLR7 and the aMPV vaccine construct modeled using Pymol.

Molecular docking was performed between the aMPV construct and a Toll-like receptor (TLR7) to analyze the potential immunogenic ability of the multiepitope vaccine. As previously discussed, TLR7 was selected from the ten Toll-like receptors due to its role as a major immune sensor, utilizing various pathways to trigger the innate immune response. Using the Uniprot database, the sequence of TLR7 was retrieved and modeled using AlphaFold. The docking analysis was done using Hdock, where the results indicate a strong binding affinity between the vaccine construct and TLR7. Figure 12 shows the visualization of the construct binding as well as the docking interaction analysis of the amino acid residues and formation of hydrogen bonds and hydrophobic interactions as predicted by the PDBsum server in Tamarind Bio.

### 3.10. In Silico Cloning and Immune Simulation of the Designed aMPV Multi-Epitope DNA Vaccine

**Figure 13:**
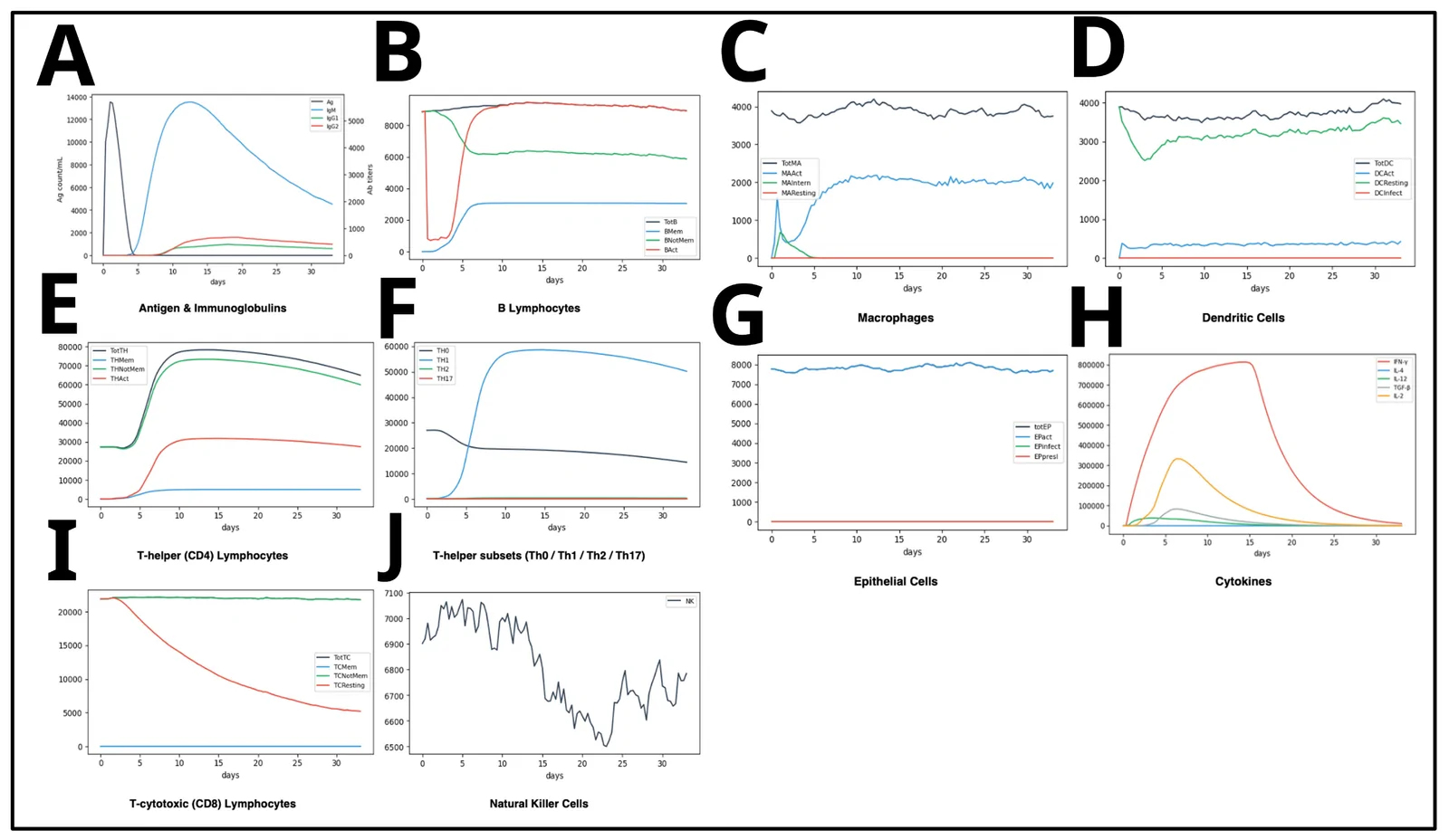
In silico immune simulation of the multiepitope aMPV DNA vaccine construct: (**A**) total counts of antigens and immunoglobulins (IgM, IgG1, IgG2); (**B**) B lymphocyte population divided by subtype; (**C**) macrophage population totals and by state (**D**) dendritic cell counts; (**E**) T-helper lymphocyte counts; (**F**) subset of T-helper cell counts (Th0, TH1, TH2, Th17); (**G**) epithelial cell counts across various states; (**H**) cytokine profiles during the simulation period; (**I**) cytotoxic T lymphocytes by state; (**J**) natural killer cell population over time. The simulation was performed utilizing the antigen-combined sequence data from two proteins (F and G) of avian metapneumovirus, and it was set to 100 with a volume of 20 mL.

**Figure 14:**
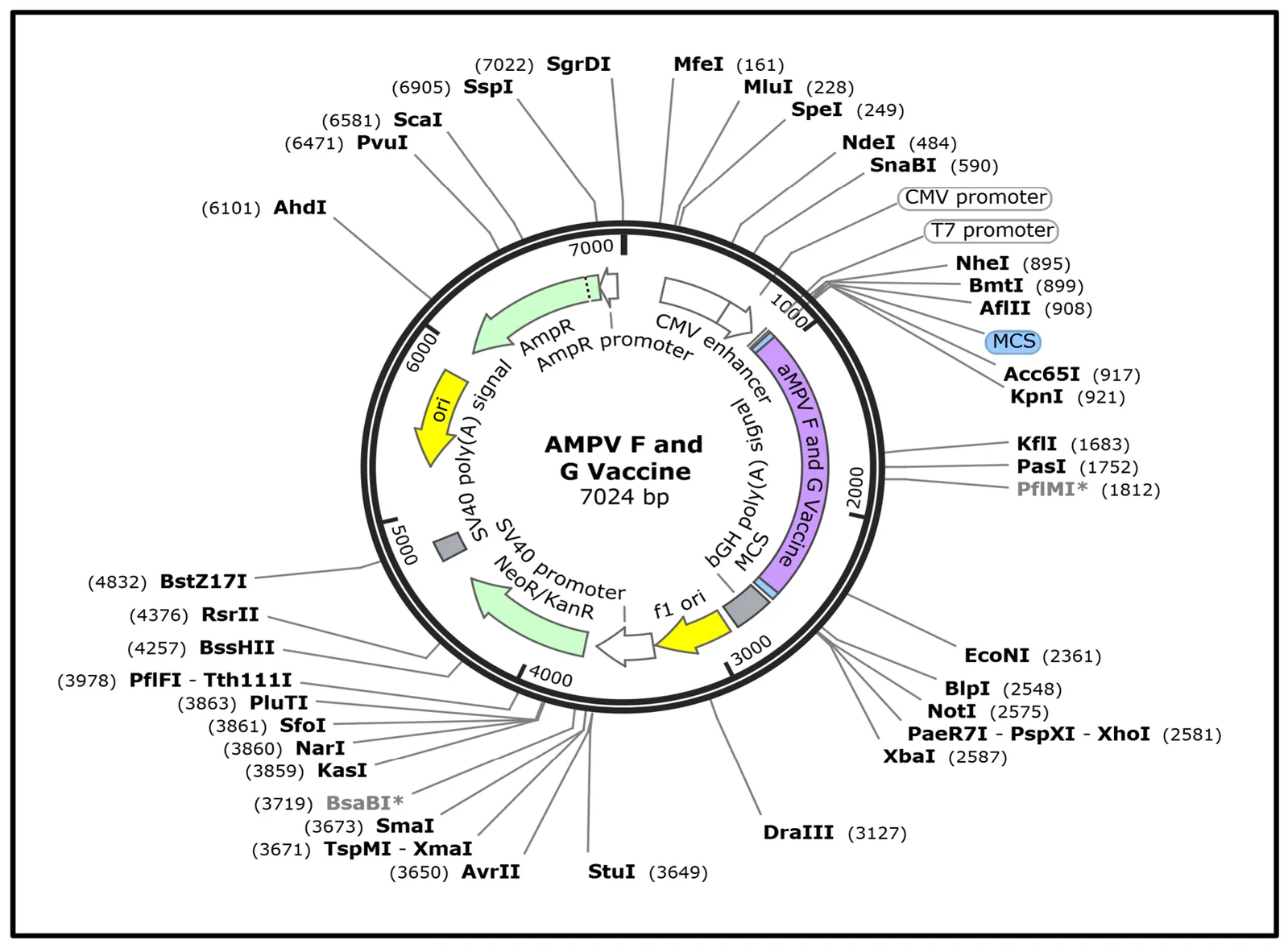
The Vector map showing the in silico cloning of the multiepitope (F and G) aMPV vaccine construct into pcDNA3.1(+) expression vector.

Shown in Figure 13 is the simulated immune response of the vaccine as seen through the B cell, T cell, and cytokine interactions. For cloning of the vaccine construct, the amino acid sequence was decoded using VectorBuilder for the expression within expression vector pcDNA3.1(+). The vector was cloned on SnapGene between the restriction enzyme cutting locations of BamHI and EcoRI, as seen in Figure 14. Supplementary Figure S1 shows the immune simulation without IL-18 in the construct.

## 4. Discussion

Avian metapneumovirus is a major emerging respiratory virus, with significant impact on the global poultry industry and with a morbidity capable of affecting up to 10% of the population [1]. Many of the current vaccines for aMPV reduce clinical conditions and viral dissemination, but not all are equally effective in the prevention of infection and circulation, allowing for continued viral persistence of the virus [2]. This scenario triggers an urgent need for the development of effective vaccines that can cover currently circulating subtypes across the globe. As described in previously published articles, the use of machine learning has greatly improved, with multiepitope vaccines showing a long-term protection through induced trained immunity, but by also being safer and cost-effective [9,10,11,18,19].

In this study, the aim was to use a machine learning immunoinformatic approach to design a multiepitope-based DNA vaccine, targeting two important proteins for avian metapneumovirus, F and G proteins. Using 53 isolates for the F protein and 68 isolates for the G protein, the vaccine construct targets the circulating isolates across the globe to provide maximum coverage. The vaccine construct was developed, showing promising physiochemical properties as well as high antigenicity and a tertiary structure suitable for further work and analysis. Using immune simulation, the designed aMPV vaccine construct was able to demonstrate a high potential for propagating humoral and cellular immune responses in silico. The data reflect a sequence conservation, seeming to have a potential advantage to be tested in vivo. Our results show that this vaccine construct potentially induces robust antibody production with class switching as well as a pertinent cytotoxic T cell response, lasting T-helper cell response, and immune activation seen by the cytokine profile.

The pipeline of the design and in silico cloning of the multiepitope DNA vaccine is as follows: (1) retrieval of the amino acid sequences, (2) multiple sequence alignment generating a consensus sequence, (3) prediction of B cell (linear and discontinuous) and T cell epitopes (MHC class I and II), (4) molecular docking of T cell epitopes, (5) selection of top-ranked epitopes and screening against the chicken amino acid genome, (6) construction of the multiepitope vaccine using linkers, (7) the use of IL-18 as adjuvant to the construct, (8) prediction of the physiochemical properties, (9) prediction of the secondary and tertiary structures, (10) molecular docking of the vaccine construct to chicken Toll-like receptor (TLR7), and (11) immune simulation of the final vaccine construct and in silico cloning to assess the ability of the construct to elicit humoral and cellular immunity in chickens. For both B and T cells, predictions yield numerous epitopes per protein. Criteria filters were set to make sure the best epitopes were selected. All of the predicted epitopes were screened for allergenicity, antigenicity, and non-toxicity [64,65,66]. For B cell epitopes, the predicted epitopes were screened against the conformational epitopes predicted by ElliPro, wherein an overlap in residues indicates that the regions include properties from both linear and conformational B cell epitopes; an inclusion that is carried further to the multiepitope vaccine construct. In terms of the MHC II epitopes, a percentile score of <4 and IC50 < 50 nM was set as the criteria for filtering. Molecular docking was performed on all of the MHC-based epitopes, whereby any that could not dock when using the HDock server were filtered.

After the filtering, top-ranked peptides were selected based upon binding and high antigenic scores. Through the analysis of the conservation of the epitopes, we found that the F protein had 53 isolates, comprising 18 subtype A, 28 subtype B, 6 subtype C, and 1 mutant isolate, spanning over six avian hosts and five countries. For the G protein, with 68 isolates, there are 15 subtype A, 49 subtype B, 3 subtype C, and 1 mutant isolate, spanning over five avian hosts and five countries. It was then identified that two epitopes (RRKGFGIL and VRRKGFGIL) within the F protein have a 100% sequence identity across the 53 isolates. This shows the conservation that bridges the three subtypes and the mutant throughout all six avian species and the five countries listed. On the other hand, the G protein epitopes show an exclusive specificity towards subtype B due to the high hypervariability of the G protein. Three epitopes, ICISVEQV, SICISVEQV, and CISVEQVKL, have a 100% sequence identity to the subtype B isolates used, showing exceptional coverage. Of the targeted lineages, the used epitopes in the vaccine have crossed the host species barrier covering commercial poultry lines such as chickens and turkeys across international boundaries, based upon the computational analysis, to be further verified by in vivo testing. This conservation could theoretically minimize the risk of strain escape, for which further testing would be needed. The epitopes were constructed into the vaccine using linkers and adjuvants. For this vaccine, chicken IL-18 was selected as the adjuvant. It was selected based upon its importance as a cytokine in the innate and adaptive immune immunity, as well as it being used in various vaccine constructs against other viruses such as IBDV, NDV, and H5 influenza [53,54,67,68]. For the preservation of conformational epitope conformation, flexible linkers were used, like GPGPG and EAAK. The 3D structure prediction was completed with AlphaFold, and a Ramachandran plot analysis along with other structural analysis were done to confirm proper folding and that the construct is solvent accessible.

A challenge presented in the study is that chicken alleles are not available in the IEDB server for MHC class I and II prediction. Alternative strategies were used to overcome this issue, using things such as published binding motifs for MHC class I epitopes [45,46]. Specifically, MHC I has very little computational tools available, so that the use of human surrogate models was not possible. For MHC class II, the human surrogate model was able to be used based upon previous work done looking into the groove homology between human and chicken alleles, leaving the default settings for the predictions. With this prediction, MixMHC2pred was able to be used to predict the corresponding chicken alleles (Gaga_BLB1_002_01, Gaga_BLB1_012_01, Gaga_BLB2_002_01, Gaga_BLB2_012_01, Gaga_BLB2_012_02) [50]. These values, like % rank and IC50, are taken at a computational standpoint as a proof-of-concept as they are still human based.

To predict the presence of cysteine residues containing a reactive thiol (-SH) group in the side chain that, when incorporated into the protein, would have major biochemical impact, a disulfide bond analysis was done. Disulfide bonds normally form due to the oxidation reaction between two cysteine residues, making the vaccine structure more stable. The spatial orientations of the two cysteines linked are determined by the conformational classification of the X^3^ dihedral angle (C-S-S-C), describing the rotation around the S-S bond [69]. At an angle of approximately ±90◦, bonds show either the right- or left-handed conformations, and when approaching 180◦, it corresponds to a trans conformation, showing an arrangement of cysteines that is extended and linear. When the angle is close to 0◦, this indicated a cis conformation, which is energetically unstable due to its steric clashes and unfavorable geometry. As the prediction software came up with no bonds fulfilling these criteria, no changes to the structure were made.

The normal mode analysis (NMA) was used to further validate the stability and flexibility of the vaccine construct. In the mobility plot, residues with higher peaks (0.5–2 arbitrary units) correspond to flexible loops or linker regions, while lower values (0–0.3 arbitrary units) correspond to stable structural regions. Bending during motion is shown in the deformability plot in the range of 0.05–0.15. The B-factor plot assesses atomic fluctuations where values below 50 indicate stable proteins and values above 50 indicate flexible proteins. Eigenvalues in the range of 1 × 10^−6^ to 1 × 10^−4^ are representative of the stiffness of a protein, whereby the vaccine protein at the higher end of this range shows good structural rigidity and a significant amount of energy would be needed for deformation. The variance plot shows individual (purple) and cumulative (green) variances, with lower individual variance values (<0.05) representing the restricted motion of each mode, while the accumulation of modes was represented by a rise in the cumulative curve. Lastly, the stable regions seen with dense springs and flexible areas with sparse springs make up the elastic network.

In another study, a multiepitope vaccine candidate for infectious bronchitis virus in silico exhibited a strong binding to both TLR3 and TLR7 [70]. Following this, TLR7 was used for molecular docking. TLR3 was not used as it recognizes double-stranded RNA while aMPV is single-stranded. TLR7 is a primary endosomal receptor targeting single-stranded RNA viruses like aMPV. The receptor is present on various cell types such as plasmacytoid dendritic cells, B cells, eosinophils, neutrophils, and monocytes/macrophages, making it a crucial target for therapeutic research such as that associated with vaccine development. Previous studies have been published on similar multiepitope vaccines using TLR7 docking for viruses such as the Newcastle disease virus and the avian leukosis virus, where strong binding and likely robust interactions were seen in the proposed virus and TLR7. The docking simulation to TLR7 exhibited a docking score between the receptor and the vaccine construct of -294.71.

The last part of the study included an in silico immune simulation of the vaccine construct on the C-ImmSim server. The C-ImmSim is a very important server that provides predictive insights into the immunogenicity of the vaccine construct. The server is also a major limitation in this study as it is optimized for human (mammalian) immune parameters. Distinct anatomical and physiological immune profiles such as the absence of encapsulated lymph nodes, unique splenic microenvironments, and highly specialized major histocompatibility complex profiles are seen in avian species. As the C-ImmSim server cannot replicate these features, the results are not a guarantee that the vaccine can provide absolute protective immunity for avian species. These results are in silico, pointing rather towards a proof of concept and confirmation of a theoretical processing capacity, one which can be treated as a baseline for required in vivo validation in avian models.

In silico modeling suggests simulated expansion of T cell populations such as cytotoxic T cells and helper T cells, all theoretical components of humoral and cellular immunity. This simulation supports the construction of a cloning candidate as done using VectorBuilder and SnapGene. From this analysis the vaccine construct has a simulated potential to elicit humoral and cellular immunity and should be confirmed through further studies with in vivo validation in avian models, which in turn could change the role aMPV has, protecting chickens and other birds from infection. The early generations of DNA vaccines have important disadvantages, including the low level of vaccine delivery to the target cells, which impacts the production of the immune response in the host after vaccination [71,72,73,74]. The new generations of DNA vaccines have several modifications to overcome these problems, including encapsulation with some nanoparticles and the conjugation of some immune stimulators, particularly chicken IL-18 [20,54,75]. To improve the efficacy of this designed multiepitope DNA vaccine against aMPV, this vaccine construct should be encapsulated with some nanoparticles. This encapsulation will enhance the delivery of the vaccine into the target cells, which will potentiate the immune response. This approach will overcome the low expression problem of the early generation of DNA vaccines or those DNA vaccines that lack any nanoparticle [71,72,73,74]. Some recent studies suggesting incorporation of IL18 in the DNA vaccine construct against Newcastle diseases virus potentiate humoral and cell-mediated immunity suggesting its positive impact as a vaccine adjuvant [20,54,75]. When looking at the simulation with IL-18, there is a stronger antibody titer overall, suggesting an enhanced predicted humoral response and indicating minimal differences in simulation results. There are many unique criteria of the aMPV vaccine construct designed in this study, including the following: (1) this vaccine construct potentially offers protection against aMPV based upon the observed conservation; (2) the vaccine construct will be encapsulated with nanoparticles which enhance the delivery of the vaccine into the target cells; (3) the vaccine includes chicken IL18 as an adjuvant, which further enhances the immune response of the vaccinated host; (4) this vaccine could potentially protect multiple species of birds, particularly chickens and turkeys, allowing for multispecies protection based upon conservation, to be validated by further testing; (5) there is the possibility to update these vaccines to match circulating strains in case of any genomic-level changes to the virus; and (6) the ability to offer potential mass vaccination of this designed vaccine, including in ovo administration. All of these criteria make the designed new vaccine against aMPV an attractive vaccine for the poultry industry not only in the US but worldwide.

It is important to note that the use of human-derived platforms like C-IMMSIM and NetMHCIIpan can affect predictive performance, indicating a strong need for further studies. For future studies, the vaccine construct should be experimentally validated, homing in on the study of the protein’s stability, safety and initial efficacy. Following that, further studies into in vivo evaluation can show the vaccine construct in a more complex system with a harder look at the safety and effectiveness of the immune response. Dosing can then be tested to see how the product performs against various strains of aMPV, allowing for testing in animals such as chickens, turkeys, and ducks. In the event of more isolates, the construct can be updated using immunoinformatics to streamline all changes including, but not limited to, new epitopes and property testing.

## 5. Conclusions

A multiepitope DNA-based vaccine candidate for aMPV was designed in silico, utilizing 18 top-ranked epitopes based upon immunogenicity, non-allergenicity, and non-toxicity. The epitopes included B cell, MHC class I, and MHC class II from major surface proteins in the virus (F and G). Structural analysis indicated strong predicted binding affinity of the T cell epitopes to chicken alleles. The selected epitopes were linked together and had chicken IL-18 attached as an adjuvant. Interaction modeling showed a favorable binding affinity between the vaccine construct and Toll-like receptor 7. The vaccine was successfully cloned in silico and when undergoing computational immune simulation, showed high immunogenic potential in both humoral and cell-mediated immunity. The vaccine construct through computational analysis establishes itself as a candidate for protection against various circulating strains of aMPV. It is important to note the design of the vaccine is based upon computational prediction tools when assessing the high potential of the vaccine in terms of immunogenicity and structural behaviors. These characteristics are bound to vary in biological systems due to various factors such as the role in the cell, processing of the proteins, and modifications made post translation. Future work is proposed to validate the multiepitope vaccine construct in vitro, working towards animal models for full usage.

## Figures and Tables

**Table 1:** The computational tools used in the study and their information.

Computational Tool	Primary Training Dataset/Database	Species Basis	Target Application for Study
AlphaFold Server	Protein Data Bank (PDB), Uniprot	Universal (all taxa)	3D modeling of vaccine construct
trRosetta	Protein Data Bank (PDB)	Universal (all taxa)	3D structure of viral proteins
PSIPRED	Protein Data Bank (PDB)	Universal (all taxa)	Secondary structure of vaccine construct
DEEPTMHMM	PDBTM, OPM, Uniprot, and SignalP 6.0	Universal (all taxa)	Membrane topology predictions of viral proteins
ProtParam (Expasy)	UniProtKB/Swiss-Prot database	Universal (chemical)	Computation of molecular weight, pI, and stability index of vaccine construct
ProtScale (Expasy)	Kyte and Doolittle hydropathy scale	Universal (chemical)	Grand average of hydropathicity (GRAVY) of viral proteins
Innovagen Solubility Calculator	Hydropathy index algorithms	Universal (chemical)	Water solubility of vaccine construct
Disulfide by Design	RCSB Protein Data Bank (PDB)	Universal (structural)	Engineering stabilizing disulfide bridges for vaccine construct
ProSA-web server	Protein Data Bank (PDB)	Universal (structural)	Overall structural quality validation (Z-score) of vaccine construct
Ramplot	Worldwide Protein Data Bank (wwPDB)/RCSB PDB	Universal (structural)	Dihedral angle validation of vaccine construct
PDBsum	Protein Data Bank (PDB)	Universal (structural)	Binding affinity between vaccine construct and TLR7
Pymol	Geometric coordinate visualization engine	Universal	3D rendering/visualization of binding, constructs, and solvent accessibility
iMODS server	Internal coordinates normal mode analysis	Universal (physics)	Molecular dynamic simulations of vaccine construct
HDOCK	PDB protein–ligand structural complexes, RCSB Protein Data Bank	Universal (structural)	Molecular docking analysis
Geneious Prime (ToxinPred 3.0.)	Sequence data manipulation workspace	Universal (bioinformatics)	Multiple sequence alignment (MSA) of aMPV strains and phylogenetic trees
SnapGene	Multi-source cloning annotation workspace	Universal (bioinformatics)	In silico simulation of expression vector
Vector Builder	Vector framework database	Universal (bioinformatics)	Conversion of amino acid vaccine construct to nucleic acid
VaxiJen	Protective viral antigens	Pathogens	Filtering of epitopes based upon antigenicity
AllerTOP	Multi-species allergens	Multi-species	Filtering of epitopes based upon allergenicity
ToxinPred	Toxic and non-toxic peptides from Swissprot and TrEMBLE	Multi-species	Filtering of epitopes based upon toxicity
ABCPred	BCIPEP database	Multi-species	Predictions of linear B cell epitopes for aMPV viral proteins
ElliPro	3D geometrical algorithm	Universal (structural)	Prediction of discontinuous/conformational B cell epitopes for aMPV viral proteins
NetMHCIIpan	Immune Epitope Database (IEDB)	Human, mouse	Prediction of T cell epitopes for aMPV viral proteins
MixMHCpred2	Immune Epitope Database (IEDB), immunopeptidomic studies	Human, mouse, bovine, equid, gallus, ovids, primates, suids	Prediction of the chicken alleles associated with the NetMHCIIpan predicted T cell epitopes
C-ImmSim	Position-specific scoring matrix (PSSM), Miyazawa–Jernigan residue–residue potential matrix	Human	Immune simulation of vaccine construct

**Table 2:** List of the top-ranked B cell epitopes among the consensus representing the currently circulating aMPV strains in chickens based on their F and G proteins.

Starting Position	Epitope	Score	Antigen/Non-Antigen Property
F Protein			
4	KLLLIIYLVVGASGKIQE	0.77	0.6201
60	CNDGPSLISTELSLTQNA	0.76	0.563
64	PSLISTELSLTQNALQ	0.66	0.8262
68	STELSLTQNALQELRTVS	0.82	0.7314
133	EVKAIKLALRSTNEAVST	0.72	1.1422
134	VKAIKLALRSTNEAVS	0.69	1.2031
486	KSNIGAALAITILVVL	0.67	0.7249
488	NIGAALAITILVVLSMSI	0.8	0.9328
494	AITILVVLSMSIIVGIAY	0.59	0.8551
500	VLSMSIIVGIAYYVVKKR	0.83	0.755
505	IIVGIAYYVVKKRKAK	0.73	0.7763
507	VGIAYYVVKKRKAKAKNG	0.86	0.7168
**G Protein**			
201	NSSQGEQPRREPTSSN	0.79	0.4667
203	SQGEQPRREPTSSNTTIA	0.65	0.4989
259	NHKTNTRGTSKTTDQRPR	0.76	0.7643
315	VNPREGGISPTAIQKN	0.85	1.627
324	PTAIQKNPTTQSNLVD	0.82	0.5394
339	DCTLSDPNEPPRICYQVG	0.83	0.8212
353	YQVGTYNPSQSGTCNI	0.79	0.4244
376	YGYACMATLYDTPFNC	0.91	0.6155
391	CWRRTRRCICDSGGELIE	0.79	0.6961

**Table 3:** List of the predicted conformational/discontinuous B cell epitopes among the consensus representing the currently circulating aMPV strains based on the F and G proteins.

No.	Peptide	No. of Residues	Score
F Protein			
1	A:K527, A:T528, A:T529, A:G530, A:Q531, A:S532, A:N533, A:M534, A:G535, A:Y536, A:I537, A:S538	12	0.988
2	A:A521, A:K522, A:N523, A:G524, A:Y525, A:P526	6	0.971
3	A:D479, A:L480, A:D482, A:I483, A:E484, A:V485, A:K486, A:S487, A:N488, A:I489, A:G490, A:A491, A:A492, A:L493, A:A494, A:I495, A:T496, A:I497, A:L498, A:V499, A:V500, A:L501, A:S502, A:M503, A:S504, A:I505, A:I506, A:V507, A:G508, A:I509, A:A510, A:Y511, A:Y512, A:V513, A:V514, A:K515, A:K516, A:R517, A:K518	39	0.825
4	A:E51, A:I52, A:G53, A:N54, A:V55, A:E56, A:N57, A:I58, A:T59, A:C60, A:N61, A:D62, A:G63, A:P64, A:S65, A:L66, A:I67, A:S68, A:T69, A:E70, A:L71, A:S72, A:L73, A:T74, A:Q75, A:N76, A:A77, A:L78, A:Q79, A:E80, A:L81, A:R82, A:T83, A:V84, A:S85, A:A86, A:D87, A:I89, A:T90, A:K91, A:N93, A:R94, A:A123, A:A125, A:K126, A:T127, A:I128, A:R129, A:L130, A:E131, A:G132, A:E133, A:V134, A:K135, A:A136, A:I137, A:K138, A:L139, A:A140, A:L141, A:R142, A:S143, A:T144, A:N145, A:E146, A:A147, A:V148, A:S149, A:T150, A:L151, A:G152, A:N153, A:G154, A:V155, A:R156, A:I157, A:N163, A:L165, A:K166, A:E167, A:F168, A:I169, A:S170, A:K171, A:K172, A:L173, A:T174, A:P175, A:A176, A:I177, A:N178, A:Q179, A:N180, A:K181, A:C182, A:N183, A:I184, A:A185, A:D186, A:I187, A:R188, A:M189, A:A190, A:I191, A:S192, A:F193, A:G194, A:Q195, A:N196, A:N197, A:L201, A:R205, A:A211, A:G261, A:V262, A:Y263, A:G264, A:G265, A:T266	119	0.704
5	A:M1, A:Y2, A:L3, A:K4, A:L5, A:L6, A:L7, A:I8, A:I9, A:Y10, A:L11, A:V12, A:V13, A:G14, A:A15, A:S16, A:G17, A:K18, A:I19, A:Q20, A:E21, A:T22, A:E25, A:E26, A:T32, A:R33, A:D331, A:S371, A:P377, A:L378, A:G379, A:C384, A:Y385, A:E386, A:G387, A:V388, A:S389, A:C390, A:S391, A:I392, A:G393, A:S394, A:N395, A:K396, A:V397, A:G398, A:I399, A:I400, A:K401, A:Q402, A:L403, A:N404, A:K405, A:G406, A:T408, A:H409, A:I410, A:P411, A:N412, A:N413, A:E414, A:A415, A:D416, A:T417, A:I418, A:T419, A:I420, A:D421, A:N422, A:T423, A:I424, A:Y425, A:Q426, A:L427, A:S428, A:K429, A:V430, A:V431, A:G432, A:E433, A:Q434, A:R435, A:T436, A:I437, A:K438, A:G439, A:A440, A:P441	88	0.691
**G Protein**			
**1**	A:K261, A:T262, A:N263, A:T264, A:R265	5	0.977
**2**	A:R256, A:T257, A:P258, A:N259, A:H260	5	0.949
**3**	A:G266, A:T267, A:S268, A:K269, A:T270, A:T271, A:D272, A:Q273, A:R274, A:P275, A:R276	11	0.939
**4**	A:Q354, A:V355, A:G356, A:T357, A:Y358, A:N359, A:P360, A:S361, A:Q362, A:S363, A:G364, A:T365, A:C366, A:N367, A:I368	15	0.882
**5**	A:T277, A:G278, A:P279, A:T280, A:P281, A:E282, A:R283, A:P284	8	0.819
**6**	A:K372, A:C373, A:S374, A:T375, A:Y376, A:G377, A:Y378, A:A379, A:C380, A:M381, A:A382, A:T383, A:L384, A:Y385, A:D386, A:T387, A:P388, A:F389, A:R394	19	0.805
**7**	A:T188, A:P189, A:P190, A:V191, A:H192, A:S193, A:D194, A:R195, A:A196, A:S197, A:K198, A:E199, A:H200, A:N201, A:S202, A:S203, A:Q204, A:G205, A:E206, A:Q207, A:P208, A:R209	22	0.798
**8**	A:I351, A:C352, A:Y353, A:E369, A:V370, A:P371	6	0.775
**9**	A:E211, A:P212, A:T213, A:S214, A:S215, A:N216, A:T217, A:T218, A:I219, A:A220, A:G221, A:T222, A:P223, A:S224, A:T225, A:E226, A:D227, A:T228, A:A229, A:K230	20	0.724
**10**	A:N390, A:C391, A:W392, A:R393, A:T395, A:R396, A:C398, A:I399, A:C400, A:D401, A:S402, A:G403, A:G404, A:E405, A:L406, A:I407, A:E408, A:W409, A:C410, A:C411, A:T412, A:S413, A:Q414, A:N415, A:W416	25	0.706
**11**	A:S243, A:K244, A:R245, A:G246, A:S247, A:S248, A:S249, A:S250, A:T251, A:K252, A:A253, A:A254, A:S255	13	0.636
**12**	A:G320, A:G321, A:I322, A:S323, A:P324, A:T325, A:A326, A:I327, A:Q328, A:K329, A:N330, A:P331, A:T332, A:T333, A:Q334, A:S335, A:N336, A:L337, A:V338, A:D339, A:C340, A:T341, A:L342, A:S343, A:D344, A:P345, A:N346, A:E347, A:P348, A:P349	30	0.615
**13**	A:Q60, A:C61, A:V62, A:D63, A:T64, A:Y65, A:W66, A:A67, A:E68, A:N69, A:G70, A:S71, A:L72, A:H73, A:P74, A:G75, A:Q76, A:S77, A:T78, A:E79, A:N80	21	0.599
**14**	A:R285, A:Q286, A:T287, A:H288	4	0.54
**15**	A:K86, A:T87, A:T88, A:T89, A:K90, A:D91, A:P92	7	0.521

**Table 4:** List of the top-ranked predicted MHC class I epitopes for aMPV sequences based on the F and G proteins.

Protein	Chicken Allele	Peptide	Antigenicity Score
F	BF2*12	GGTVVYMV	0.5041
		LAITILVV	1.1036
		LLLIIYLV	0.5608
		TTAAVTAGV	0.8088
		AALAITILV	0.8116
		IIVGIAYYV	0.6574
		KLLLIIYLV	0.4294
		VLGAIALGV	1.1038
		KGFGILIGV	0.5864
		LLLIIYLVV	0.4445
	BF2*19	CRHERESYA	0.8337
		VRAINRMP	0.4295
		HRKKRFVL	1.0261
		RRKGFGIL	2.1777
		VRRKGFGIL	1.5903
	BF2∗02:01/BF2∗04:01/BF2∗21:01	AALAITILV	0.8116
		AAVTAGVAL	0.8072
		VALAKTIRL	0.7919
		VALTPLGGL	0.748
		GAALAITIL	0.7037
		LAITILVVL	0.9243
		VVLSMSIIV	0.6253
		IVGIAYYVV	0.6651
		LVVLSMSII	0.8192
		VVNNFNPLL	0.6228
		EVKAIKLAL	0.9369
		IIVGIAYYV	0.6574
		NIADIRMAI	1.3016
		QITKENRIL	0.6728
		ALAITILVV	0.9803
		KLLLIIYLV	0.4294
		LLLIIYLVV	0.4445
		VLGAIALGV	1.1038
		ILVVLSMSI	0.7834
		ELSLTQNAL	0.8262
		YSEESCSTV	0.4523
		LSHRKKRFV	1.463
		ISKKLTPAI	0.8982
		LSMSIIVGI	1.0499
		VSMVALTPL	1.4032
		KGFGILIGV	0.5864
		IGVYGGTVV	0.7106
		LGVATTAAV	0.9871
		IGAALAITI	0.9508
		AGVALAKTI	0.7927
		FGQNNRRFL	0.9863
		AGITSAVSL	0.6628
G	BF2*12	ICISVEQV	1.7104
		ELYTIEGV	0.4977
		SSEIVLKQV	0.5542
		EPPRICYQV	0.8991
	BF2*19	LRRSKRIL	0.6266
		RRSKRILL	0.6951
		LRRSKRILL	0.5764
		KRILLGLVL	0.583
	BF2∗02:01/BF2∗04:01/BF2∗21:01	QVLRRSKRI	0.7462
		AVLGGVDCL	0.846
		SICISVEQV	1.4046
		CISVEQVKL	1.5918
		VLRRSKRIL	0.5308
		LLGLVLSAL	0.5858
		GLVLSALGL	0.93
		VLSALGLTL	1.1061
		EPPRICYQV	0.8991
		SSEIVLKQV	0.5542
		PSQSGTCNI	0.4112
		RSKRILLGL	1.1368
		YGYACMATL	0.7578

**Table 5:** The criteria of the predicted MHC class II epitopes for the aMPV F and G proteins.

Protein	Allele	Chicken Allele	Peptide	IC50 < 50 nM from NetMHCII	Per Rank % from NetMHCII	Antigenicity Score
F	HLA-DRB1*13:66	Gaga_BLB1_002_01	QISLMLNNRAMVRRK	27.14	0.05	0.6937
			ISLMLNNRAMVRRKG	37.77	0.18	0.607
		Gaga_BLB2_002_01	SGQISLMLNNRAMVR	29.21	0.05	0.6209
G	HLA-DRB1*14:82	Gaga_BLB2_012_01	QVLRRSKRILLGLVL	29.28	0.01	0.6683

**Table 6:** List of the selected aMPV-B and T cell epitopes incorporated in the aMPV multiepitope DNA vaccine construct.

S.No	Protein	Start	Peptide	Antigenicity Score	Docking Score	Confidence Score
B Cell Epitopes						
1	F	60	CNDGPSLISTELSLTQNA	0.6201		
2		68	STELSLTQNALQELRTVS	0.7314		
3		133	EVKAIKLALRSTNEAVST	1.1422		
4		134	VKAIKLALRSTNEAVS	1.2031		
5		259	NHKTNTRGTSKTTDQRPR	0.7643		
6	G	315	VNPREGGISPTAIQKN	1.627		
7		339	DCTLSDPNEPPRICYQVG	0.8212		
8		376	YGYACMATLYDTPFNC	0.6155		
MHC Class I Epitopes						
9	F	96	LSHRKKRFV	1.463	−235.5	0.8468
10		251	VRRKGFGIL	1.5903	−212.17	0.7762
11		252	RRKGFGIL	2.1777	−204.96	0.7501
12	G	48	SICISVEQV	1.4046	−186.24	0.6737
13		49	ICISVEQV	1.7104	−151.07	0.5053
14		50	CISVEQVKL	1.5918	−202.06	0.7391
MHC Class II Epitopes						
15	F	238	SGQISLMLNNRAMVR	0.6209	−185	0.6682
16		240	QISLMLNNRAMVRRK	0.6937	−204.61	0.7488
17		241	ISLMLNNRAMVRRKG	0.607	−221.83	0.8079
18	G	20	QVLRRSKRILLGLVL	0.6683	−217.95	0.7956

## Data Availability

The raw data supporting the conclusions of this article will be made available by the authors upon request.

## Supplementary Materials

The following supporting information can be downloaded at https://www.mdpi.com/article/10.3390/v18101084/s1, File S1. 55 F Protein isolates of aMPV; File S2. G Protein Isolates of aMPV; File S3. List of the predicted MHC class I epitopes for aMPV and their docking scores predicted from the Hdock server; File S4. List of the predicted MHC class II epitopes for aMPV and their docking scores predicted from the Hdock server; File S5. Selected Epitopes Consensus to Original Sequences; Figure S1. Immune simulation of the vaccine constrict without the IL-18 adjuvant.

## Abbreviations

The following abbreviations are used in this manuscript:

**Abbreviation**	**Full name**
aMPV	Avian metapneumovirus
MHC-I	Major histocompatibility-I
MHC-II	Major histocompatibility-II
F	Fusion protein
G	Attachment protein
IL-18	Interleukin-18
TLR	Toll-like receptors
