# Supplementary material for: Integrative Machine Learning and Immunoinformatics-Guided Design and In Silico Validation of a Multiepitope DNA Vaccine Against Avian Metapneumovirus
Source: Viruses. 2026 Oct 1;18(10):1084. doi: 10.3390/v18101084 (PMC13709672; doi:10.3390/v18101084)
Supplement: Supplementary file 1 [file viruses-18-01084-s001.zip › aMPV Supplemental Data/Supplementary Figures.pdf]

Supplementary Figures:

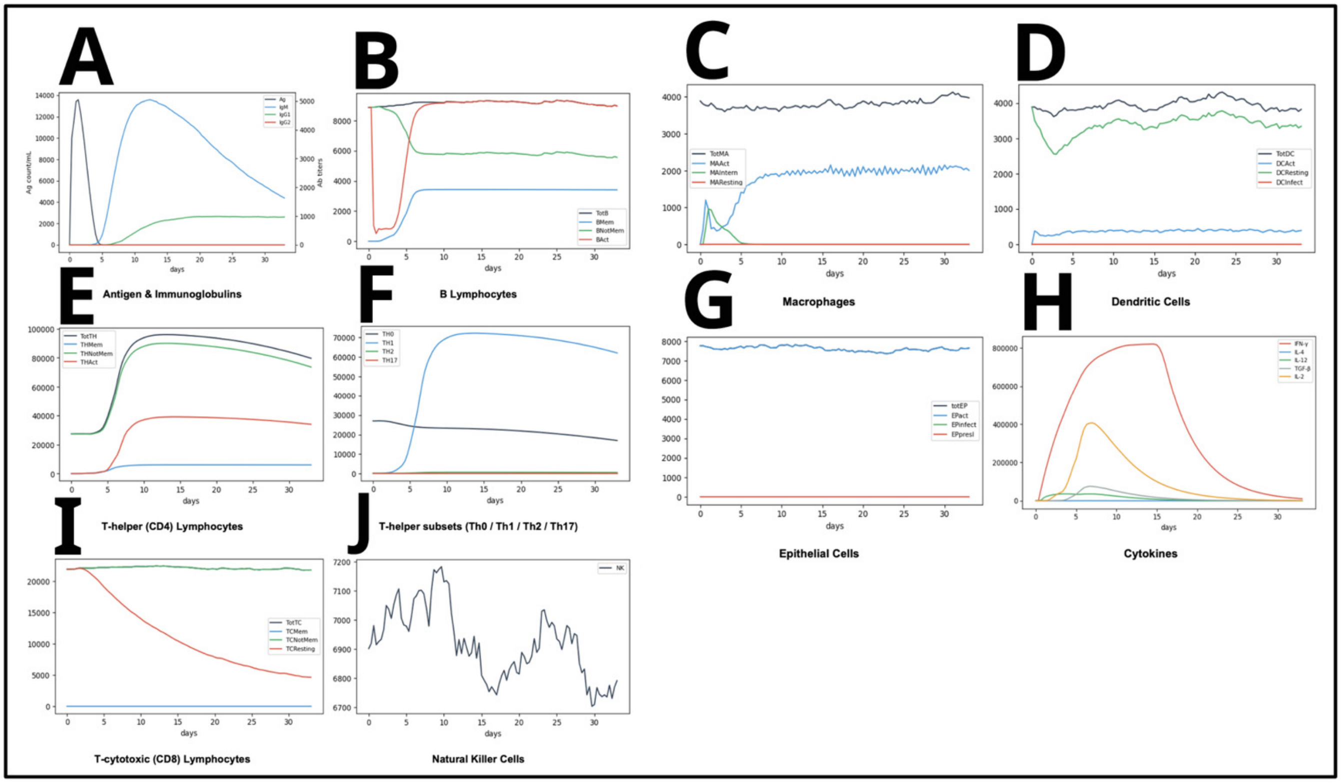

Supplementary Figure S1. Immune simulation of the vaccine constrict without the IL-18 adjuvant.
